# Screening and selection of essential oils for an intranasal spray against bovine respiratory pathogens based on antimicrobial, antiviral, immunomodulatory, and antibiofilm activities

**DOI:** 10.3389/fvets.2024.1360398

**Published:** 2024-02-07

**Authors:** Samat Amat, Gabriela Magossi, AGM Rakibuzzaman, Devin B. Holman, Kaycie N. Schmidt, Luke Kosel, Sheela Ramamoorthy

**Affiliations:** ^1^Department of Microbiological Sciences, North Dakota State University, Fargo, ND, United States; ^2^Lacombe Research and Development Centre, Agriculture and Agri-Food Canada, Lacombe, AB, Canada; ^3^Department of Biological Sciences, North Dakota State University, Fargo, ND, United States

**Keywords:** bovine respiratory pathogens, essential oil, antimicrobial, antiviral and antibiofilm activities, immunomodulation, nasopharyngeal microbiota

## Abstract

**Introduction:**

The rise in antibiotic resistant pathogens associated with bovine respiratory disease (BRD) poses a serious challenge, particularly to the beef feedlot industry, as they currently depend on antibiotics to prevent BRD to mitigate the financial burden (approx. $1 billion annual loss) inflicted by BRD-associated high mortality and morbidity in feedlot cattle. Thus, there is an impetus need for the development of antimicrobial alternative strategies against BRD. This study aimed to screen and select candidate essential oils (EOs) for the development of an intranasal EO spray that can inhibit BRD pathogens and promote microbiota-mediated respiratory health.

**Methods:**

The effects of selected EOs (ajowan, cinnamon leaf, citronella, grapefruit, fennel, and thyme) on a bovine nasopharyngeal microbiota culture were evaluated using 16S rRNA gene sequencing. The microbiota culture was enriched by incubating nasopharyngeal swabs obtained from finishing beef heifers in brain heart infusion broth with and without EOs (0.025%, v/v). These EOs were then also evaluated for their immunomodulatory effects on bovine turbinate (BT) cells by analyzing the concentrations of 15 cytokines and chemokines in cell culture after 24 h incubation. The crystal violet assay was done to assess the antibiofilm activity of EOs against *Escherichia coli* UMN026 strain. Finally, 15 EOs were screened for their antiviral activity against the bovine viral diarrhea virus 1 (BVDV-1) using BT cells and a fluorescence-based method.

**Results:**

Ajowan, fennel, and thyme resulted in a moderate reduction of overall nasopharyngeal microbiota growth with significant alterations of both alpha and beta diversity, and the relative abundance of predominant bacterial families (e.g., increasing *Enterobacteriaceae* and decreasing *Moraxellaceae*) compared to the control (*p* < 0.05). Co-incubation of BT cells with selected EOs resulted in minimal alterations in cytokine and chemokine levels (*p* > 0.05). Ajowan, thyme, fennel, and cinnamon leaf exhibited antibiofilm activity at concentrations of 0.025 and 0.05%. Reduction of BVDV-1 replication in BT cells was observed with thyme (strong), and ajowan and citronella (moderate) at 0.0125% concentration.

**Discussion:**

Accordingly, ajowan, thyme, fennel, cinnamon leaf, and citronella EOs were selected for further development as an intranasal EO spray to prevent and control of BRD pathogens in feedlot cattle.

## 1 Introduction

Bovine respiratory disease (BRD) is a complex infectious process resulting from the interplay of various factors, including environmental conditions, the immune status of the animal, and the presence of bacterial and viral agents (1). The primary bacterial pathogens associated with BRD are *Mannheimia haemolytica, Pasteurella multocida, Histophilus somni*, and *Mycoplasma bovis* (2). In addition to bacteria, viruses also play a significant role in BRD, including bovine herpesvirus-1 (BHV-1), bovine viral diarrhea virus (BVDV), bovine respiratory syncytial virus (BRSV), parainfluenza-3 (PI-3), and bovine respiratory coronavirus (BRCV) (3).

Feedlot cattle, particularly those newly feedlot arrivals are susceptible to BRD due to the numerous stress factors, such as weaning, transportation, changes in diet, contact with other animals, and concurrent diseases (4). These stressors can weaken the immune response of the cattle, making them more susceptible to viral infections and disrupting respiratory microbiota homeostasis. This disruption can then predispose animals to pneumonia due to the translocation of pathogenic bacteria from the upper to lower respiratory tract (1, 5). Despite extensive prevention and treatment efforts, including vaccination and antibiotic administration, BRD morbidity and mortality rates persist or even increased, which is partially due to the rise in antimicrobial resistance (AMR) (4, 6–8). Recent research shows that more than 50% of bovine respiratory pathogens harbor AMR levels that can exceed 50% and can often display multidrug resistance (9–12), which limits antibiotic effectiveness, particularly against multidrug-resistant strains. Therefore, manipulating the microbiota to restore respiratory homeostasis presents a promising approach for enhancing respiratory resilience against BRD (13).

Essential oils (EOs) are aromatic compounds extracted from plants and can exhibit antimicrobial activity against bacteria and viruses (14–16). These characteristics make them potential natural antibiotic alternatives. Developing antimicrobials that can inhibit pathogen growth without collateral damage to the commensal microbiota is an alternative approach to mitigating infectious diseases. The EOs of ajowan (AJO), cinnamon leaf (CIN), citronella (CIT), fennel (FEN), and thyme (THY), among others, were previously characterized *in vitro* for their antimicrobial activity against the BRD-associated bacterial pathogens *M. haemolytica*, *P. multocida*, and *H. somni* (17–19). Studies also suggest that EOs may modify antimicrobial resistance, potentially reverting multidrug-resistant bacteria to a susceptible state when used alongside antibiotics (20–23). Moreover, EOs have been shown to possess antiviral activity against other respiratory viral pathogens (24–26). For example, an EO blend consisting of three EOs inhibited influenza A (H1N1) and herpes simplex virus 1 as well as the bacterial pathogens (methicillin-resistant *Staphylococcus aureus, Streptococcus pneumoniae,* and *Klebsiella pneumoniae*) *in vitro*, suggesting potential for treating influenza and post-influenza bacterial pneumonia (27). Additionally, some EOs have been reported to have antifungal (27), antibiofilm (28, 29), and immunomodulatory properties (30). All of which makes EO-based strategy as an appealing antimicrobial alternative approach to mitigate BRD in feedlot cattle. The objective of this study was to screen and select candidate EOs for development of an intranasal EO spray against bacterial and viral pathogens associated with BRD in feedlot cattle, as an alternative to antibiotics. The screening criteria included the effect of EOs on bovine nasopharyngeal swab (NS) microbiota culture, antiviral activity against bovine respiratory viral pathogens, antibiofilm activity against *Escherichia coli*, and immunomodulatory activities.

## 2 Materials and methods

**Figure 1:**
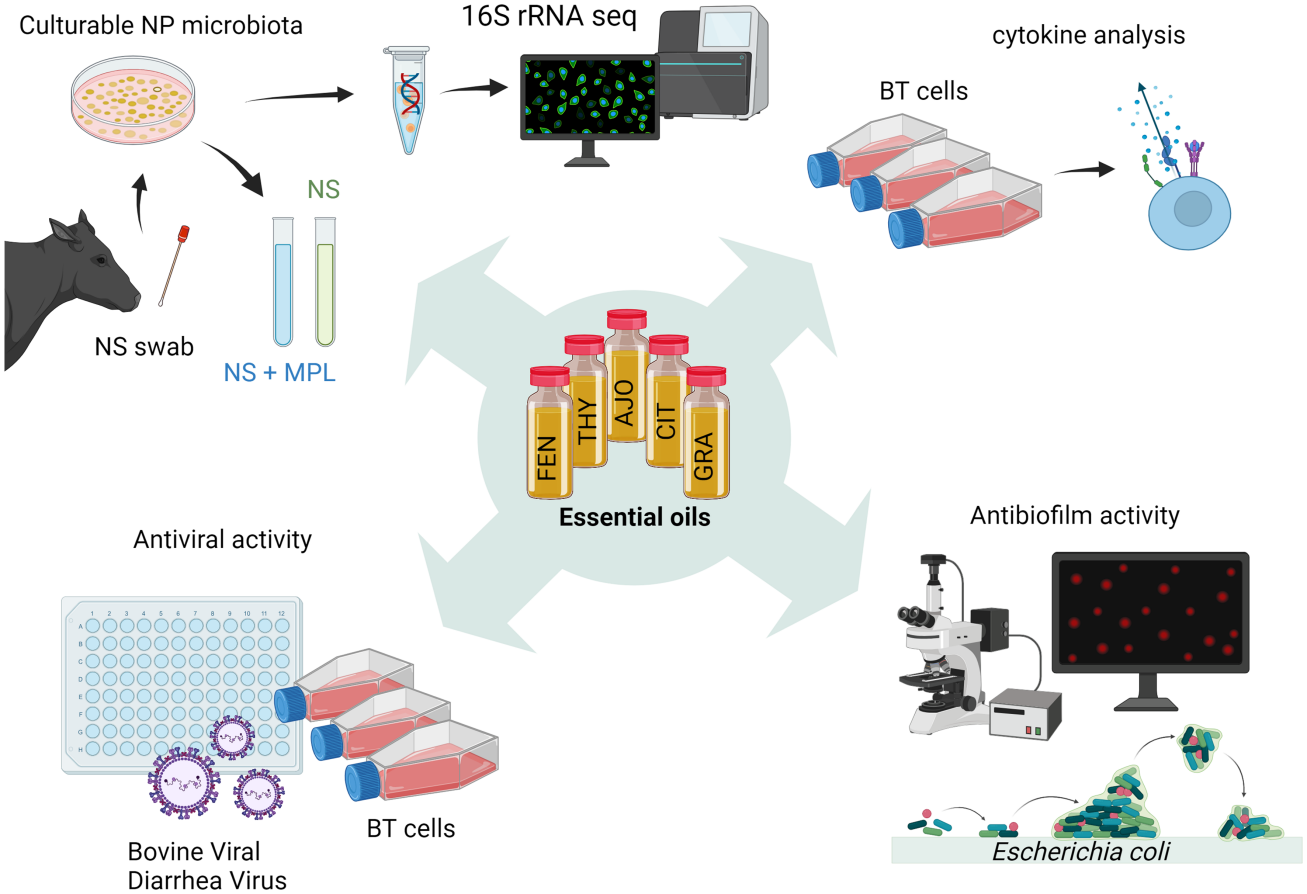
Schematic workflow diagram illustrating the process of screening essential oils (EOs) *in vitro* (created with BioRender.com).

We previously identified minimum inhibitory concentrations (MICs) and minimum bactericidal concentrations of 15 EOs against *M. haemolytica, P. multocida*, and *H. somni* (18). Among those 15 EOs, five EOs were selected for this study and these selected EOs include AJO, THY, FEN (MIC ≤ 0.025%), CIN, and CIT (MIC ≤ 0.05%). The selected EOs displayed greater inhibition against all three BRD pathogens as compared to the remaining 9 EOs. In the present study, we further evaluated these five selected EOs for their antimicrobial, antiviral, immunomodulatory, and antibiofilm activities (Figure 1). Of note, we have previously tested 15 EOs including those EOs studied in the present study for their cytotoxicity on BT cells using a lawn assay, and none of these EOs exhibited any cytotoxicity within the tested range of concentrations (0.0125–0.4%, v/v) (18).

### 2.1 Antimicrobial activity of EOs against BRD-associated bacterial pathogens

First, we evaluated the effects of AJO, THY, FEN, CIN, and grapefruit (GRA) EOs on the growth of completely different *M. haemolytica* and *P. multocida* strains (obtained from the Veterinary Diagnostic Lab, North Dakota State University, Fargo, ND, United States) than those strains used in our previous study (18), as well as on the overall growth of NS microbiota using the broth macrodilution method as described previously (18). Of note, the GRA EO was included in this study as a negative control as it did not inhibit the growth of *M. haemolytica* at the maximum tested concentration of 0.4% (18). While the CIT EO was selected for this study, and was further characterized for its immune modulation, antibiofilm and antiviral activities described below, this EO was not included in the present antimicrobial activity tests as it displayed similar antimicrobial activity against BRD pathogens and the commensal bacteria as with CIN EO (18) plus the limited budget availability for 16S rRNA gene sequencing.

For the evaluation of antimicrobial activity against *M. haemolytica* and *P. multocida*, 25 μL of each EO was diluted in 975 μL dimethyl sulfoxide (DMSO, Sigma-Aldrich, St. Louis, MO, United States). Then a 50 μL aliquot of EO dissolved in DMSO was added to 5 mL brain heart infusion broth (BHI; BD, Franklin Lakes, NJ, United States) followed by inoculation with 50 μL of each of the 18 h culture of *M. haemolytica* or *P. multocida* containing 1 to 2 × 10^8^ colony forming units (CFU) per ml. The mixture was incubated aerobically at 37°C with agitation at 200 rpm for 24 h. Controls included DMSO (1%, v/v) without EOs and a negative control with no DMSO or EO added. After incubation, the optical density (OD_600_) of each bacterial culture was measured.

### 2.2 Effects of EOs on the culture-enriched bovine nasopharyngeal microbiota

To investigate the impact of EOs on the overall growth of the culture-enriched bovine NS microbiota *in vitro*, NS swab samples collected from a cohort of crossbred finishing beef heifers (*n* = 31; initial BW = 494 ± 10 (SE) kg) were used. Animal housing, handling, and feeding conditions were described elsewhere (31). Deep nasopharyngeal swabs were collected from the right nostril of each heifer using a long, guarded swab with a rayon tip (27 cm long, MW 124, Medical Wire & Equipment, Corsham, United Kingdom) as described previously (31). Upon collection, NS swabs were stored in 1 mL BHI broth containing 20% glycerol, flash frozen with dry ice and stored at −80°C. From these NS swab samples stored in BHI/glycerol, 5 samples were randomly selected and vortexed vigorously for 1 min to release and dispense microbial cells attached to the swab. Then, 200 μL of the media containing NS microbial cells from each swab were pooled. One hundred microliters of the pooled sample were then inoculated into 900 μL BHI broth and incubated at 37°C with agitation at 200 rpm for approximately 3 h until the cell density reached an OD_600_ of 0.2. From this 3 h culture, 100 μL was inoculated into 5 mL BHI broth containing 0.025% of each EO (AJO, THY, FEN, CIN, and GRA) or DMSO or no EO (negative controls) and incubated for 24 h under the same conditions as described above. To test whether the presence of the NS commensal microbiota would influence the antimicrobial activity of EOs against *M. haemolytica* and *P. multocida* or a mixture of both species, 50 μL of an overnight (18 h) culture was added to 5 mL BHI containing NS microbiota culture and an EO and incubated for 24 h. A 50 μL aliquot of an overnight (18 h) culture of *Lactobacillus fermentum* (ATCC-9338, American Type Culture Collection, Manassas, VA, United States) culture was also added along to the *M. haemolytica* or *P. multocida* cultures. Of note, *Lactobacillus* spp. present in the nasopharynx of beef cattle are beneficial and can inhibit colonization of respiratory pathogens and induce positive modulation of nasopharyngeal microbiota in feedlot cattle (13, 32, 33). Therefore, *L. fermentum* was added as a representative of the beneficial bacteria, and to identify whether EOs could inhibit the beneficial bacteria in the presence of nasopharyngeal microbiota. After incubation, the overall cell growth of each NS microbiota culture was measured at an OD of 600 nm, and 1 mL of each 24 h culture was taken and stored at −80°C for genomic DNA extraction.

### 2.3 Genomic DNA extraction from nasopharyngeal microbiota cultures

Genomic DNA was extracted from the NS microbiota culture using the Qiagen DNeasy Blood and Tissue kit (Qiagen Inc., Germantown, MD, United States) according to the manufacturer’s instructions with modifications as outlined in our previous paper (34). Briefly, 100 μL of a 24 h NS microbiota culture was pelleted by centrifugation (13,000 × *g* for 5 min), and the supernatant was removed, and then the pellet was resuspended in 180 μL of lysis buffer (20 mM Tris–HCl, pH 8.0, 2 mM sodium EDTA, and 1.2% Triton X-100) containing 300 U/mL of mutanolysin and 20 mg/mL of lysozyme. The resuspended pellet in lysis buffer was vortexed and incubated at 37°C for 1 h with agitation at 800 rpm. Then, 25 μL of proteinase K and 200 μL of buffer AL (provided in the kit, no ethanol added) were added, and the mixture was incubated at 56°C for 30 min with shaking at 800 rpm. Silica/zirconia beads (0.1 mm) were then added to the tubes (approximately 400 mg) and samples were mechanically lysed at 6.0 m/s for 20 s in a FastPrep-24 classic bead beater (MP Biomedicals, Irvine, CA, United States). Samples were then centrifuged at 13,000 × *g* for 5 min and 400 μL of the supernatant was transferred to the columns provided by the kit and from this step onward the instructions from the kit were followed.

### 2.4 16S rRNA gene sequencing and analysis

The 16S rRNA gene amplification and sequencing were carried out by Molecular Research LP (MRDNA; Shallowater, TX, United States) using the 515F (5′-GTGCCAGCMGCCGCGGTAA-3′) and 806R (5′-GGACTACHVGGGTWTCTAAT-3′) primers targeting the V4 hypervariable region, with reaction preparation and cycling conditions as described previously (35). Briefly, a 30-cycle PCR amplification was performed using the HotStarTaq Plus Master Mix Kit (Qiagen Inc., Germantown, MD, United States) and PCR products were checked for intensity and correct band size on a 2% agarose gel electrophoresis. Amplicon concentrations was normalized, indexed, pooled together, and then purified using AMPure XP beads (Beckman Coulter, Brea, CA, United States) for an Illumina DNA library preparation (Illumina Inc., San Diego, CA, United States). Sequencing was carried out in an Illumina MiSeq instrument using the MiSeq reagent kit v3 (2 × 300 bp) following the manufacturer’s instructions.

Sequences were quality filtered and processed with the DADA2 v.1.18 package (36) in R v. 4.3.1. Forward and reverse reads without primer sequences were initially trimmed to 220 bp, denoised, merged with a minimum overlap of 100 bp, and chimeras removed. The SILVA SSU database release 138.1 (37) was used to assign taxonomy to these amplicon sequence variants (ASVs) with the naïve Bayesian RDP classifier (38). Any ASVs classified as either chloroplasts, mitochondria, or eukaryota, as well as those present in the negative extraction control samples were removed from the analysis, as they were considered as contaminants. Samples were randomly subsampled to 171,000 reads prior to the calculation of richness (number of ASVs) and diversity indices (Shannon and inverse Simpson) and the Bray-Curtis dissimilarities with Phyloseq v. 1.46.0 (39) and vegan v. 2.5–7 (40) in R.

### 2.5 Immunomodulation effect of EOs on bovine turbinate cells

The selected six EOs (AJO, CIN, CIT, FEN, GRA, and THY) were evaluated for their immunomodulatory effects on BT cells. The BT cells (ATCC-1390; American Type Culture Collection) were seeded onto 6-well flat-bottom tissue culture plates at 1 × 105 cells per well and incubated using the standard culture conditions until a complete cell monolayer was achieved. The BT cell culture conditions are described previously (32). Briefly, the cell monolayer was washed with Dulbecco’s Modified Eagle Medium (DMEM, ATCC, Manassas, VA, United States), and then incubated with 2 mL of cell culture media supplemented with 0.025% EO (v/v) for 24 h. Ten microliters of each EO were diluted in 90 μL DMSO and a 25 μL aliquot of the EO dissolved in DMSO was added into 75 μL of DMEM to obtain a 2.5% EO stock solution. From each 2.5% EO stock solution, 20 μL was added to 2 mL of cell culture media. Negative controls included DMSO (0.2%, v/v) without EO and a negative control with no DMSO or EO added. After 24 h incubation, 1 mL of culture media was stored at −80°C for cytokine analysis. This experiment was repeated on three different days with three different passages of cells and each time had 2 replicates for each of EO. A total of 15 cytokines and chemokines were quantified using the Bovine Cytokine 15-Plex Discovery Assay^®^ (Millipore Sigma, Burlington, Massachusetts, United States) on the Luminex^™^ 200 instrument (Eve Technologies Corp, Calgary, Alberta, Canada) according to the manufacturer’s instructions. The 15-plex assay consisted of interferon gamma (IFN-γ), interleukins (IL) IL-1α, IL-1β, IL-4, IL-6, IL-8, IL-10, IL-17A, and IL-36RA, interferon gamma inducible protein-10 (IP-10), monocyte chemoattractant protein-1 (MCP-1), macrophage inflammation proteins (MIP) MIP-1α and MIP-1β, tumor necrosis alpha (TNFα), and vascular endothelial growth factor-A (VEGF-A). Multiplexing allowed for the simultaneous detection of the cytokines and chemokines at a sensitivity range of 0.05 to 66.51 pg./mL (Millipore Sigma MILLIPLEX^®^ MAP protocol).

### 2.6 Antibiofilm activity of EOs against *Escherichia coli*

The same selected EOs used for the immunomodulatory effects on BT cells (AJO, CIN, CIT, FEN, GRA, and THY) were also evaluated for their antibiofilm activity against *E. coli* strain UMN026 using a crystal violet (CV) biofilm assay (41). UMN026 is a clinical uropathogenic *E. coli* strain that displayed relatively strong biofilm forming capacity (P. Bergholz et al., unpublished data). For the biofilm assay, a single colony of *E. coli* UMN026 was inoculated into 200 μL of lysogeny broth (LB; BD, Franklin Lakes, NJ, United States) in a 96-well plate and incubated at 37°C for 8 h. Two microliters of this 8 h culture was then transferred to a well containing 198 μL glucose defined minimal media (GDMM) supplemented with 0.5% casamino acids (CAA; VWR, Radnor, PA, United States), prepared as described elsewhere (42), and the plate was then sealed and incubated overnight at 37°C. On the following day, 2 μL of the overnight culture was transferred to 198 μL GDMM supplemented with 0.5% CAA, incubated at 37°C for 8 h, and then 2 μL of 8 h culture was transferred to 198 μL GDMM +0.5% CAA and incubated overnight. On day 3, 2 μL of the overnight culture was transferred to 198 μL GDMM +0.5% CAA containing 0.025% or 0.05% EOs and incubated at 37°C for 48 h, to allow for biofilm formation. For EO stock solutions, 2.5% of EO stock solution was prepared by adding 25 μL of 10% EO dissolved in DMSO to 75 μL LB media. From the 2.5% EO stock solution, 2 or 4 μL was added to each well. For negative controls, DMSO (0.2%) without EO and with LB broth only was added.

After 48 h incubation, the media from the plate was removed and the well was washed using 200 μL of 1X phosphate buffered saline (PBS; Corning, Corning, NY, United States). This was repeated three times. The plate was then air dried at room temperature for 1 h. Then, 200 μL of 0.1% CV was added to the wells and incubated at room temperature for 15 min. The CV was removed by washing the wells with PBS three times, and the plate was air dried at room temperature for 1 h. Two hundred microliters of freshly prepared 80:20 ethanol:acetone solution was added to the wells and incubated at room temperature for 15 min. After incubation, 150 μL of dye solution was transferred to a new 96-well plate and the OD was measured at 580 nm using a Cytation 5 Cell Imaging Multi-Mode Reader (BioTeK, Winooski, VT, United States). For blanks, 150 μL of pure 80:20 ethanol:acetone solution was used.

### 2.7 Antiviral activity of EOs against bovine respiratory viruses

The main viral agents involved in BRD in feedlot cattle are BHV-1, BRSV, BVDV, and PI-3. Given that BVDV is often used for coinfection with BRD bacterial pathogens to experimentally induce BRD in cattle (43, 44), BVDV-1 was used as a model BRD viral pathogen for this *in vitro* screening. The 6 EOs (AJO, CIN, CIT, FEN, GRA, and THY) plus 9 more EOs [black pepper, carrot seed, eucalyptus, ginger grass, lavender, niaouli, rosemary, sandalwood, tangerine, and tea tree; further details on the origin of these EOs are provided in our previous publication (18)] that have been tested against BRD bacterial pathogens in our previous study were evaluated for their antiviral activity against BVDV-1 (ATCC-VR534; American Type Culture Collection) infection of BT cells (ATCC-1390) using a fluorescence-based assay. BT cell culturing was performed as described in our previous papers (18, 32), with minor modifications. Briefly, the BT cells were seeded onto 96-well flat-bottom tissue culture plates at 1 × 10^3^ cells per well and incubated in Dulbecco’s modified Eagle’s medium (DMEM; Thermo Fisher Scientific, Oakville, ON, Canada) supplemented with 10% fetal horse serum (ATCC) and 1X antibiotic/antimycotic solution (Penicillin–Streptomycin-Amphotericin B; Hyclone Laboratories Inc., Logan, UT, United States) at 37°C with 5% CO_2_ until 85–90% confluency was obtained. The cell monolayer was then washed three times with Hank’s Balanced Salt Solution (HBSS; Cytiva Hyclone, Marlborough, MA, United States).

Stock BVDV-1 cultures were prepared by infecting BT cell monolayers at 85–90% confluence. Following incubation for 72 h, the infected flasks were freeze-thawed three times to release cell associated virus. Stock BVSV-1 titers were determined by the standard tissue culture infective dose (TCID_50_) assay. Briefly, BT cells were seeded in a 96-well plate. When the BT cells reached 80–90% confluency in the wells, a serial dilutions of the stock BVDV-1 virus ranging from −1 to −4 was added to the BT monolayers in the wells in a volume of 100 μL, in 4 replicates per dilution, and incubated at 37°C. After 3 h of incubation, 100 μL of DMEM with 5% fetal horse serum and 1 X antibiotic/antimycotic were added to each well and incubated for at least 72 h. The plates were observed under a light microscope for any visible CPE on the BT cell sheet. The titer of the stock culture was determined and calculated using the Reed-Muench formula (45). The culture was resuspended to a titer of 1.0 × 10^4^ TCID_50_/mL in DMEM to perform the antiviral activity assay with the 15 EOs.

The antiviral activity assay was assessed as a reduction in BVDV-1 replication in the presence of EO’s, when compared to a control without EO treatment. Essentially as described before with little modification (46–48). Briefly, after BT cells seeded into 96-well plates became 80–90% confluent, the growth culture media was removed from each well and washed three times with Hank’s balanced salt solution (HBSS) (ATCC). Next, 200 μL of 1 × 10^4^ TCID_50_/ml BVDV-1 in DMEM was mixed EO to obtain at a final concentration of EO at 0.025% or 0.0125% and added to respective wells. After 3 h of incubation, fetal horse serum was added to a final concentration of 5% (v/v) to each well and incubated at 37°C with the presence of 5% CO2 for another 45 h. After 48 h incubation, the culture media was removed, and washed with HBSS and fixed with a 1:1 mixture of methanol and acetone. An immunofluorescence assay was carried out on the fixed cell sheet with a BVDV fluorescent antibody conjugate (243-FA. 1801, NVSL, Ames, IA, United States), and with 6-diamidino-2-phenylindole (DAPI; Life Technologies, Carlsbad, CA, United States). Apple-green fluorescence observed under a fluorescence microscope was indicative of BVDV-1 replication. For viral positive controls, 1 × 10^4^ TCID_50_/ml BVDV-1 in DMEM without EO or with DMSO, which was the solvent for EO, and for negative control DMEM with 5% fetal horse serum and without BVDV-1, without EO were used. The intensity of the apple-green fluorescence observed under the microscope was compared between EO-treated and control samples.

### 2.8 Statistical analysis

The effect of EO treatment on the culture-enriched NS microbiota was assessed using the Bray-Curtis dissimilarities and PERMANOVA (adonis2 function) with vegan in R. Pairwise comparisons of the Bray-Curtis dissimilarities between different EO treatment groups were done using the pairwise Adonis v. 0.4 R package with the Benjamini-Hochberg procedure used to correct *p*-values for multiple comparisons. The effect of EO treatment on alpha diversity indices (number of observed ASVs, Shannon diversity index) and the relative abundance of phyla, family, and genera of culture-enriched NS microbiota, as well as on cytokine and chemokine concentrations, and biofilm cell counts were determined using the generalized linear mixed model estimation procedure (PROC GLIMMIX) LSMEANS statement (ver. 9.4, SAS Institute Inc., Cary, NC, United States). The Shapiro–Wilk test was used to determine whether a dataset follows a normal distribution. Significance was considered at *p* < 0.05.

## 3 Results

### 3.1 Antimicrobial activity of EOs against BRD-associated bacterial pathogens

**Figure 2:**
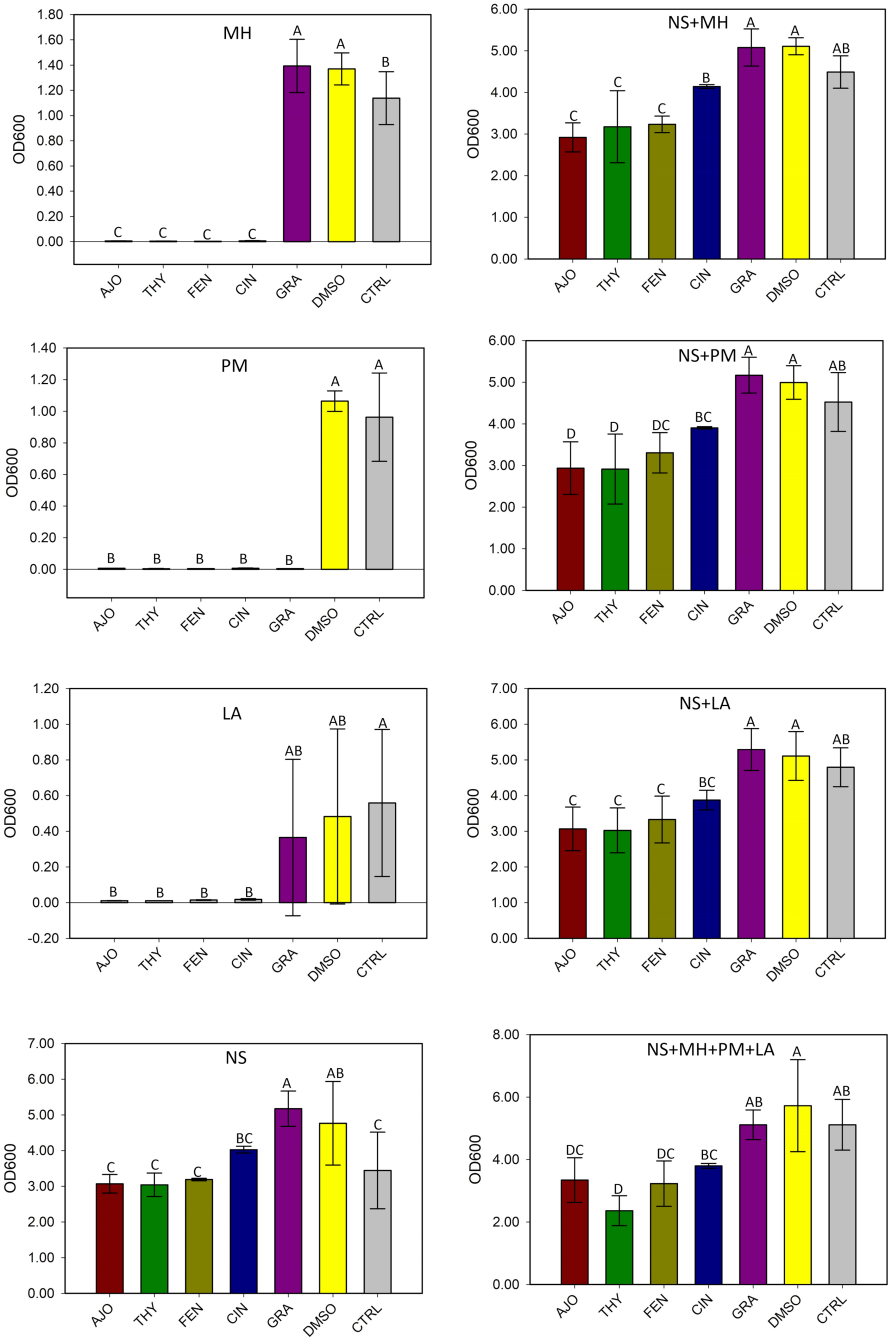
The effects of essential oils on the growth of *Mannheimia haemolytica* (MH), *Pasteurella multocida* (PM), *Lactobacillus fermentum* (LA), and the culture-enriched bovine nasopharyngeal microbiota alone (NS) or with presence of these bacteria. NS with one of the three strains (NS + MH, NS + PM, or NS + LA). NS plus all three bacterial inoculums combined (NS + MH + PM + LA). The values are the means from three replicates. Different uppercase letters indicate mean values differ (*p* < 0.05). The vertical bars indicate standard error of the mean.

The AJO, CIN, FEN, and THY EOs significantly inhibited the growth of *M. haemolytica, P. multocida*, and *L. fermentum* when compared to GRA and the negative controls, both in pure culture and in combination with the culture-enriched NS microbiota (*p* ≤ 0.05), based on the OD_600_ measurements (Figure 2).

### 3.2 Effects of EOs on the culture-enriched bovine NS microbiota

#### 3.2.1 16S rRNA gene sequencing and analysis

After processing and quality filtering, the average number of sequences per sample was 265,699 ± 60,006 (SD) (*n* = 32 samples). These sequencing reads were taxonomically assigned into 14 bacterial phyla, 94 families, and 149 genera.

#### 3.2.2 Microbial community structure, diversity, and composition

**Figure 3:**
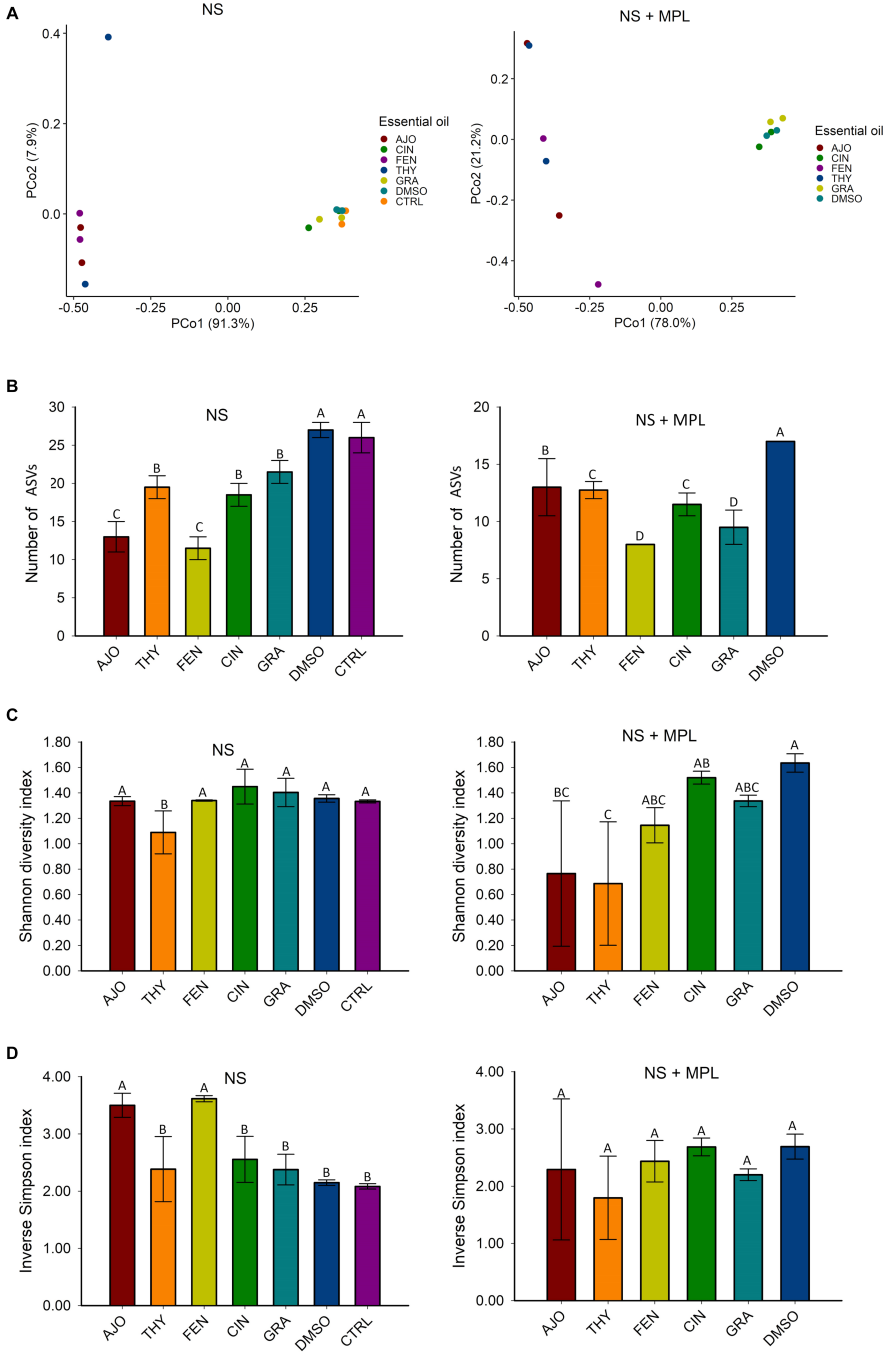
The effects of essential oils (EOs) on the beta and alpha diversities of the culture-enriched bovine nasopharyngeal swab microbiota (NS) along or with presence of *Mannheimia haemolytica, Pasteurella multocida*, and *Lactobacillus fermentum* (NS + MPL). Principle coordinates analysis (PCoA) plot of the Bray Curtis dissimilarities **(A)**, and Alpha diversity indices **(B–D)** for the enriched-NS microbiota. The values **(B–D)** are the means from two replicates. Different uppercase letters indicate mean values differ (*p* < 0.05). The vertical bars indicate standard error of the mean.

The microbial composition of the culture-enriched NS microbiota was similar between NS microbiota-only (NS) and NS with *M. haemolytica, P. multocida*, and *L. fermentum* added (MPL) samples. Although samples that were treated with the AJO, FEN, and THY clustered together and were well separated from samples that were treated with CIN, GRA, DMSO, and CTRL (Figure 3), the statistical significant difference based on PERMANOVA was not established due to the replicates in each treatment was being small (sample size = 2).

The richness of the culture-enriched NS microbiota (number of observed ASVs) was significantly different between the EO and control groups. For the NS samples, the AJO-and FEN-treated samples had the lowest number of observed ASVs, followed by CIN, GRA, and THY. Samples that were not exposed to EOs such as DMSO and control samples had the greatest richness (*p* < 0.05). For NS + MPL (Culture-enriched NS microbiota containing additional *M. haemolytica*, *P. multocida*, and *L. fermentum* inoculum) samples, the overall number of ASVs was lower than the NS samples alone, and samples treated with the FEN and GRA EOs had the lowest observed number of ASVs, followed by THY, CIN, AJO, and the DMSO control (*p* < 0.05; Figure 3).

The diversity of the NS samples as determined by the Shannon and inverse Simpson indices, did not differ between any of the EOs and the control samples, except for THY, which had a higher Shannon diversity index value (*p* < 0.05) than the others (Figure 3). Additionally, samples treated with AJO and FEN had higher inverse Simpson index value than all other groups (*p* < 0.05), including the control samples (Figure 3). Similarly, the diversity of the culture-enriched NS + MPL microbiota was similar between all the tested EOs and the control samples, except for AJO and THY, which had lower Shannon diversity index values than the remaining samples (*p* < 0.05; Figure 3).

**Figure 4:**
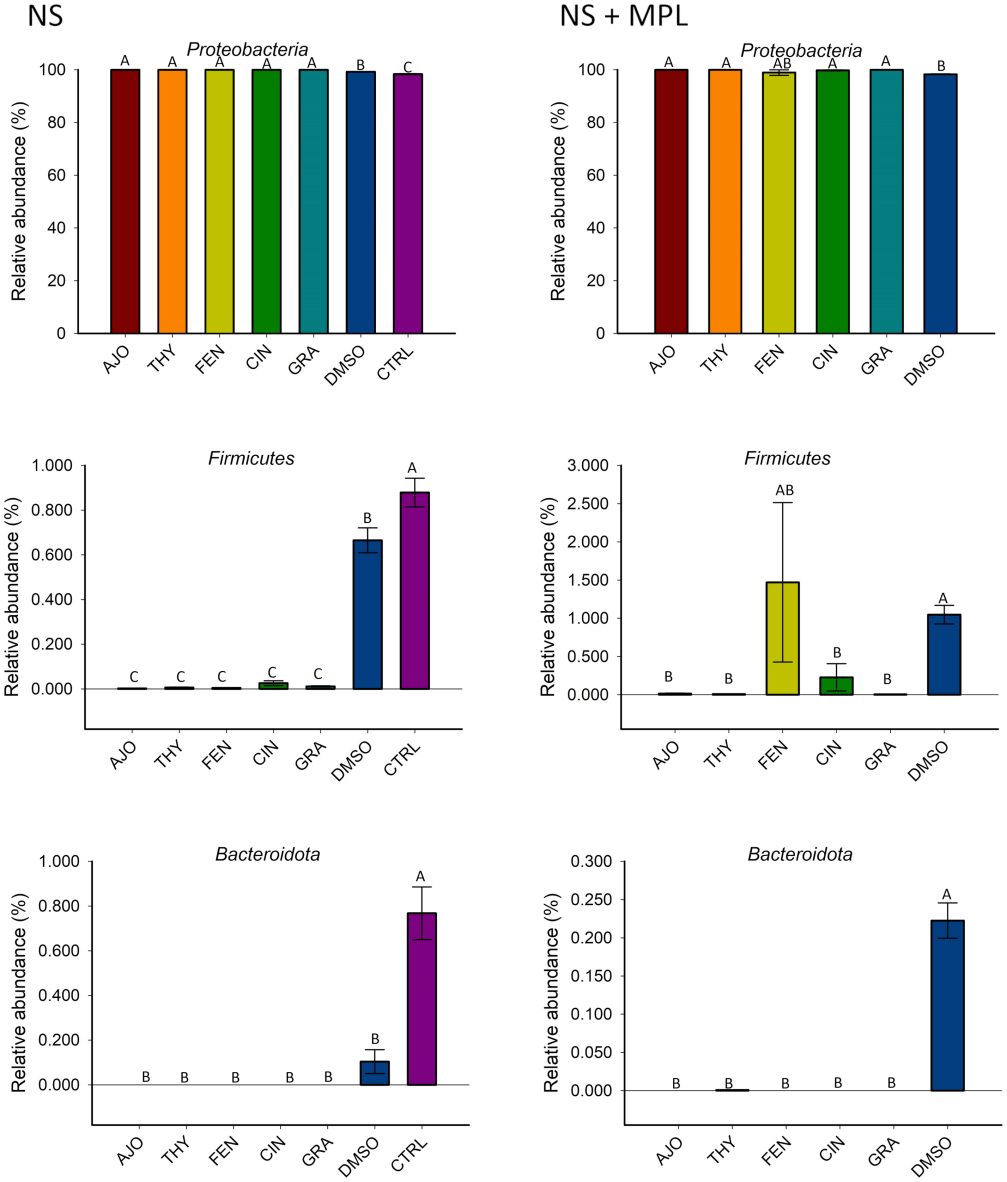
The effects of essential oils on relative abundance of the most predominant bacterial phyla in the culture-enriched bovine nasopharyngeal microbiota. The values are the means from two replicates. Different uppercase letters indicate significantly different means (*p* < 0.05).

The most relatively abundant phyla in the culture-enriched NS microbiota were *Proteobacteria* (99.59%), followed by *Firmicutes* (0.31%), and *Bacteroidota* (0.07%). The effects of EOs on the relative abundance of these three phyla were detected (Figure 4). None of the EOs tested on the NS microbiota either with (NS + MPL) or without (NS) *M. haemolytica*, *P. multocida*, and *L. fermentum* added had a significant effect on the relative abundance of *Proteobacteria*. Moreover, all EOs tested significantly reduced the relative abundance of *Firmicutes* and *Bacteroidota* in the NS microbiota when compared to DMSO or the negative control samples (*p* < 0.05). However, when *M. haemolytica*, *P. multocida*, and *L. fermentum* were added to the NS microbiota, FEN did not reduce the relative abundance of *Firmicutes* when compared to the DMSO control, as AJO, CIN, GRA, and THY did (Figure 4).

**Figure 5:**
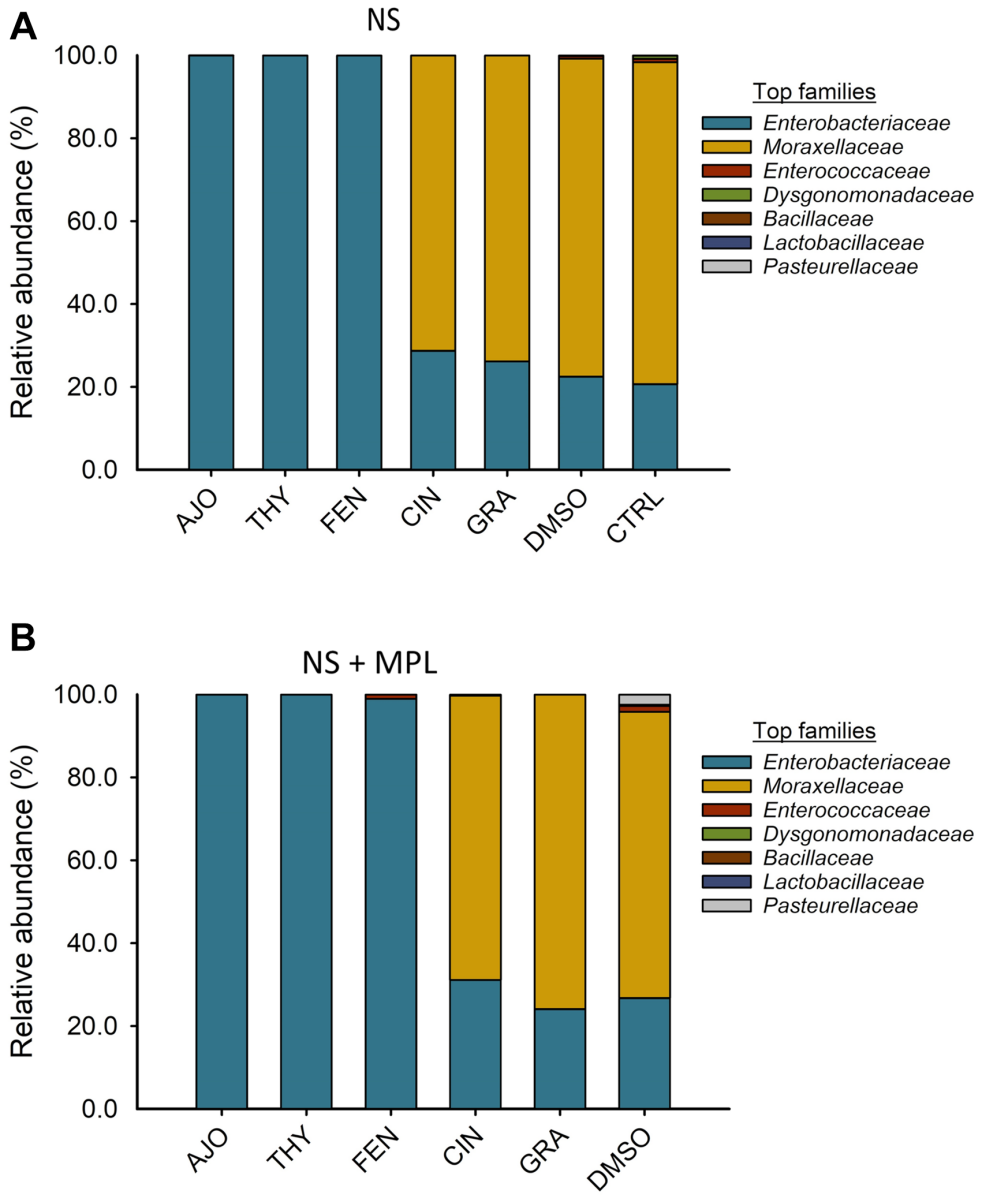
The effects of essential oils on relative abundance of the mostpredominant bacterial families present in the culture-enriched bovine nasopharyngeal microbiota with NS **(A)** or without NS  +  MPL **(B)** added *Mannheimia haemolytica*, *Pasteurella multocida*, and *Lactobacillus fermentum*. The values are the means from two replicates.

The most relatively abundant families in the culture-enriched NS microbiota were *Enterobacteriaceae* (56.9%), *Moraxellaceae* (42.8%), *Enterococcaceae* (0.21%), *Dysgonomonadaceae* (0.13%), *Bacilaceae* (0.01%), *Lactobaciliaceae* (0.005%), and *Pasteurellaceae* (0.003%). The effects of EOs on the relative abundance of bacterial families were similar between NS and NS + MPL samples. Samples treated with AJO, FEN, and THY were dominated by *Enterobacteriaceae* (>99.97%), while the most abundant families in samples treated with CIN, GRA, and DMSO were *Moraxellaceae* (65.7, 77.8, and 77.9%, respectively), followed by *Enterobacteriaceae* (34.2, 22.2%. and 21.4%, respectively; Figure 5).

**Figure 6:**
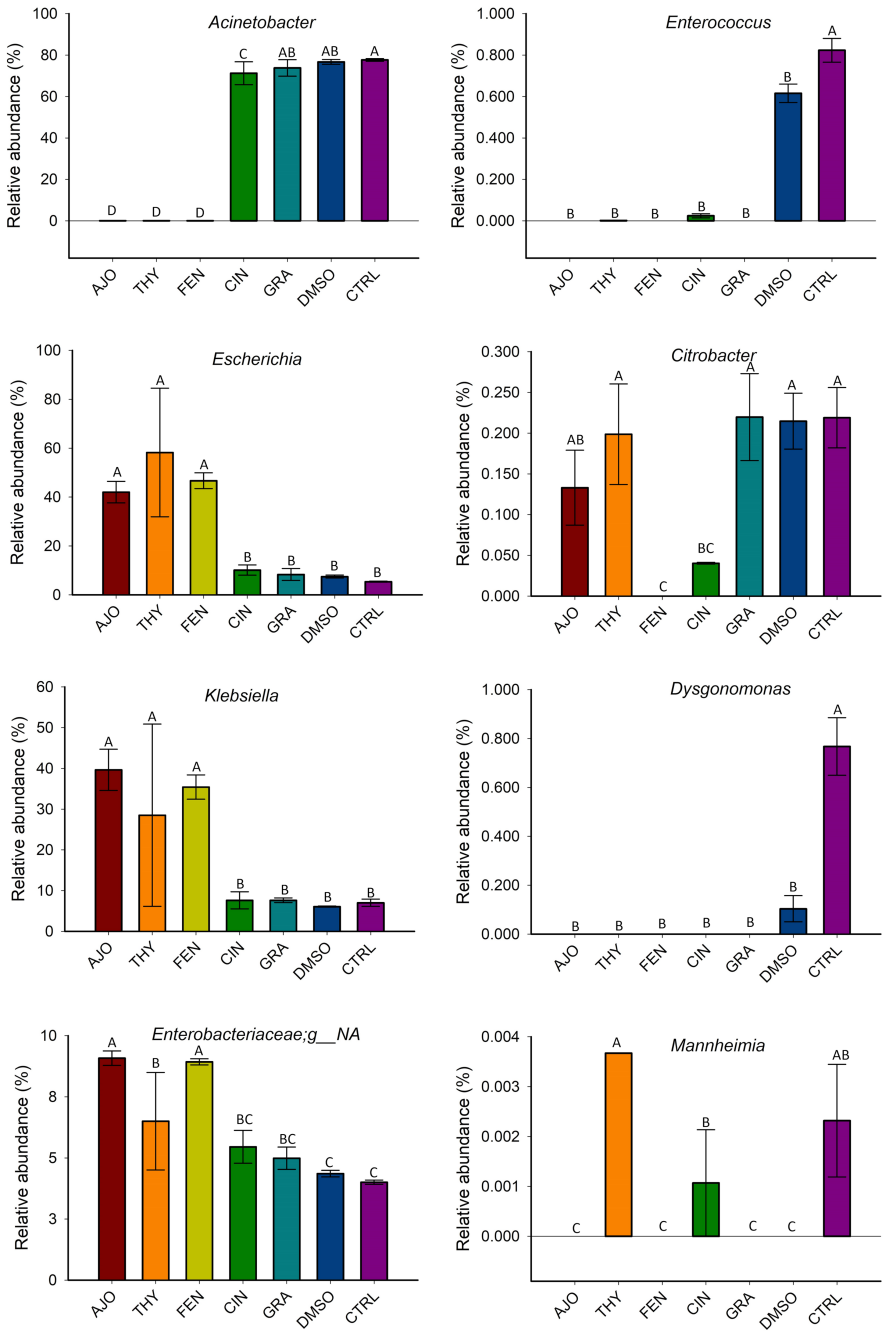
The effect of essential oils on the relative abundance of the most relatively abundant bacterial genera present in the culture-enriched bovine nasopharyngeal microbiota (NS). The values are the means from two replicates. Different uppercase letters indicate significantly different means (*p* < 0.05).

**Figure 7:**
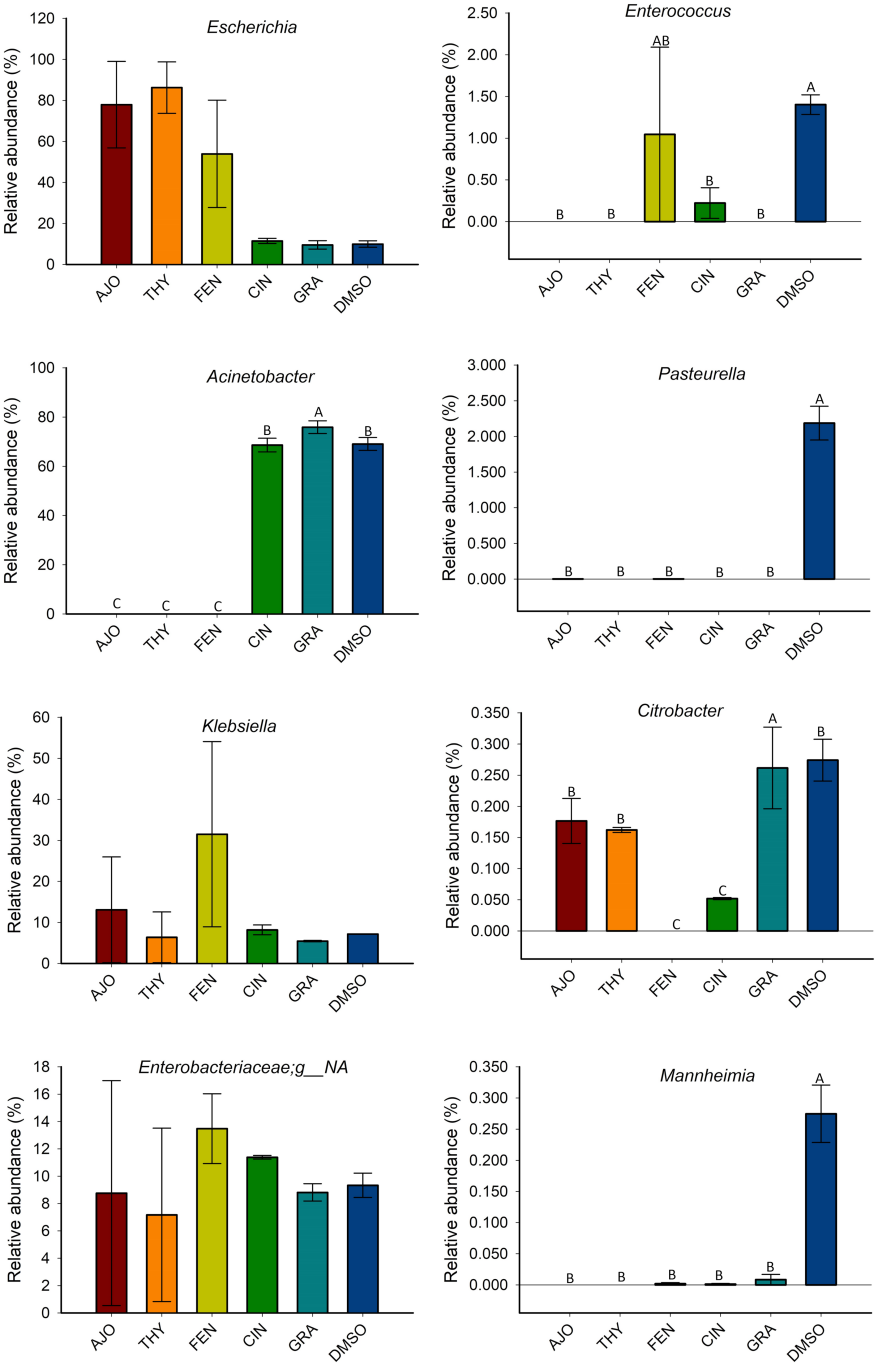
The effects of essential oils on relative abundance of the most predominant bacterial genera present in the culture-enriched bovine nasopharyngeal microbiota with *Mannheimia haemolytica*, *Pasteurella multocida*, and *Lactobacillus fermentum* added (NS + MPL). The values are the means from two replicates. Different uppercase letters indicate significantly different means (*p* < 0.05).

The most relatively abundant genera present in the culture-enriched NS microbiota (NS vs. NS + MPL) were *Acinetobacter* (42.8% vs. 35.6%), *Escherichia-Shigella* (25.5% vs. 41.5%), *Klebsiella* (18.9% vs. 12.0%), *Enterococcus* (0.21% vs. 0.45%), *Citrobacter* (0.15% vs. 0.16%), *Dysgonomonas* (0.13% vs. 0.04%), *Mannheimia* (0.01% vs. 0.05%), and *Pasteurella* (< 0.001% vs. 0.37%). The AJO, THY, and FEN EOs reduced the relative abundance of *Acinetobacter* (*p* < 0.05) and increased the relative abundance of *Escherichia-Shigella* and *Klebsiella* (*p* < 0.05) in comparison with the negative control samples (Figure 6). All EOs reduced the relative abundance of *Enterococcus* and *Dysgonomonas* (*p* < 0.05). Only FEN and CIN reduced the relative abundance of *Citrobacter* (*p* < 0.05), while AJO, FEN, GRA, and DMSO completely reduced the relative abundance of *Mannheimia* (*p* < 0.05), when compared to the control sample, in NS culture only. For the NS + MPL culture-enriched microbiota, all EOs reduced the relative abundance of *Pasteurella* and *Mannheimia* (*p* < 0.05) when compared to the DMSO control (Figure 7).

### 3.3 Immunomodulatory effects of EOs on BT cells

**Figure 8:**
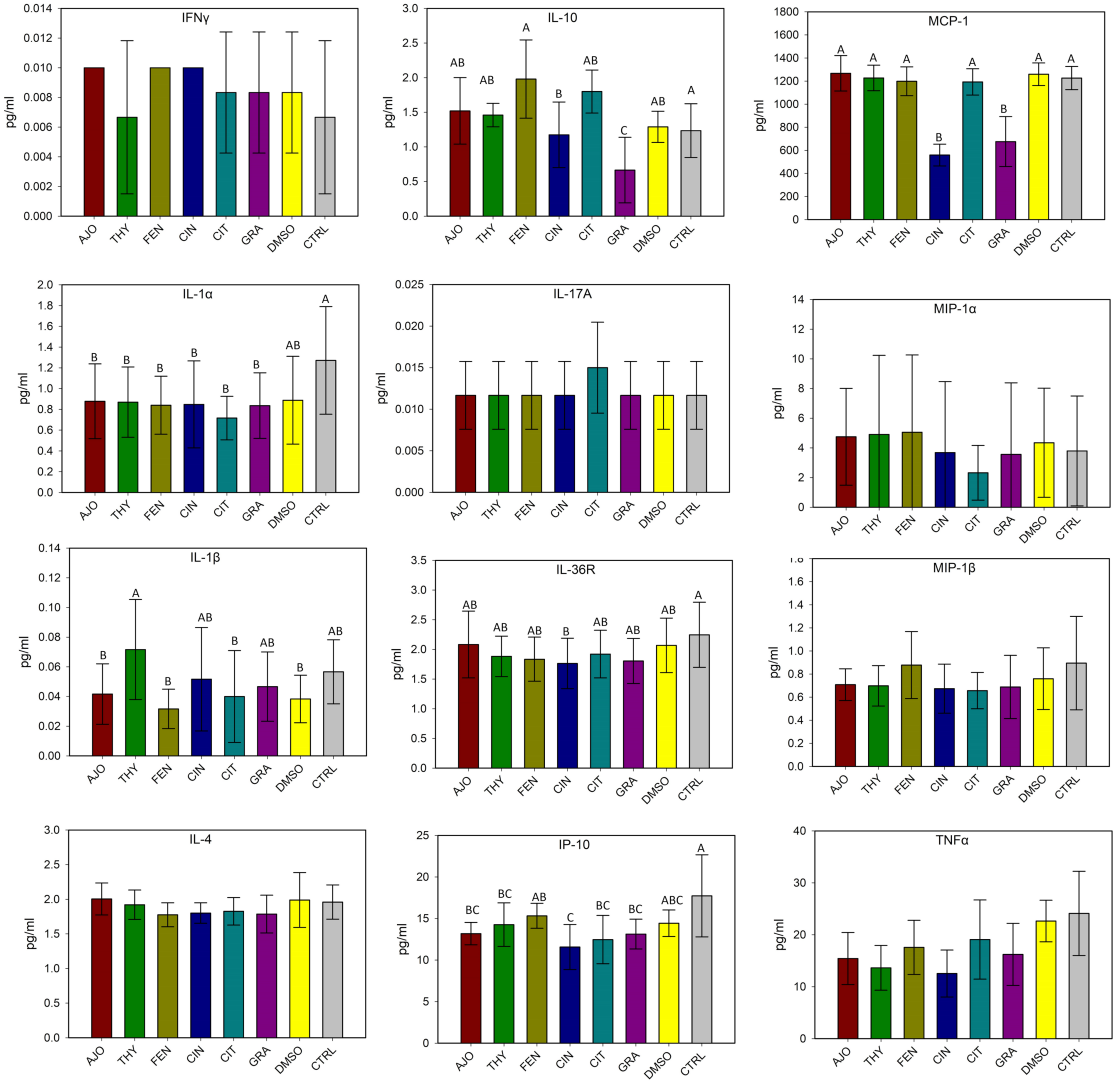
Evaluation of the effects of essential oils on cytokine production in bovine turbinate cell lines. Different uppercase letters indicate significantly different means (*p* < 0.05). The values are the means obtained from three independent experiments performed on different days. Different uppercase letters indicate significantly different means (*p* < 0.05).

The effects of co-incubation of BT cells with one of the selected EOs (AJO, THY, CIN, CIT, and GRA) for 24 h on the production of cytokine and chemokines from BT cell monolayers are presented in Figure 8. The concentrations of IFN-γ, IL-1α, IL-1β, IL-4, IL-17A, MIP-1α, MIP-1β, and TNF-α did not differ between any of the EOs tested and control groups (DMSO and CTRL) (*p* > 0.05). The concentrations of IL-36R and IP-10 were lower in BT cells co-cultured with CIN as compared to BT cells co-cultured without EO (CTRL) (*p* < 0.05). The concentration of MCP-1 was reduced in BT cells in response to co-culturing with CIN and GRA compared to the control group (*p* < 0.05). The impact of EOs on IL-6, IL-8, and VEGF-A concentrations were not established as there were significantly larger variations between replicates. Overall, co-incubation of EO for 24 h incubation resulted in minimal immune stimulation in BT cell monolayers.

### 3.4 Antibiofilm activity of EOs against *Escherichia coli*

**Figure 9:**
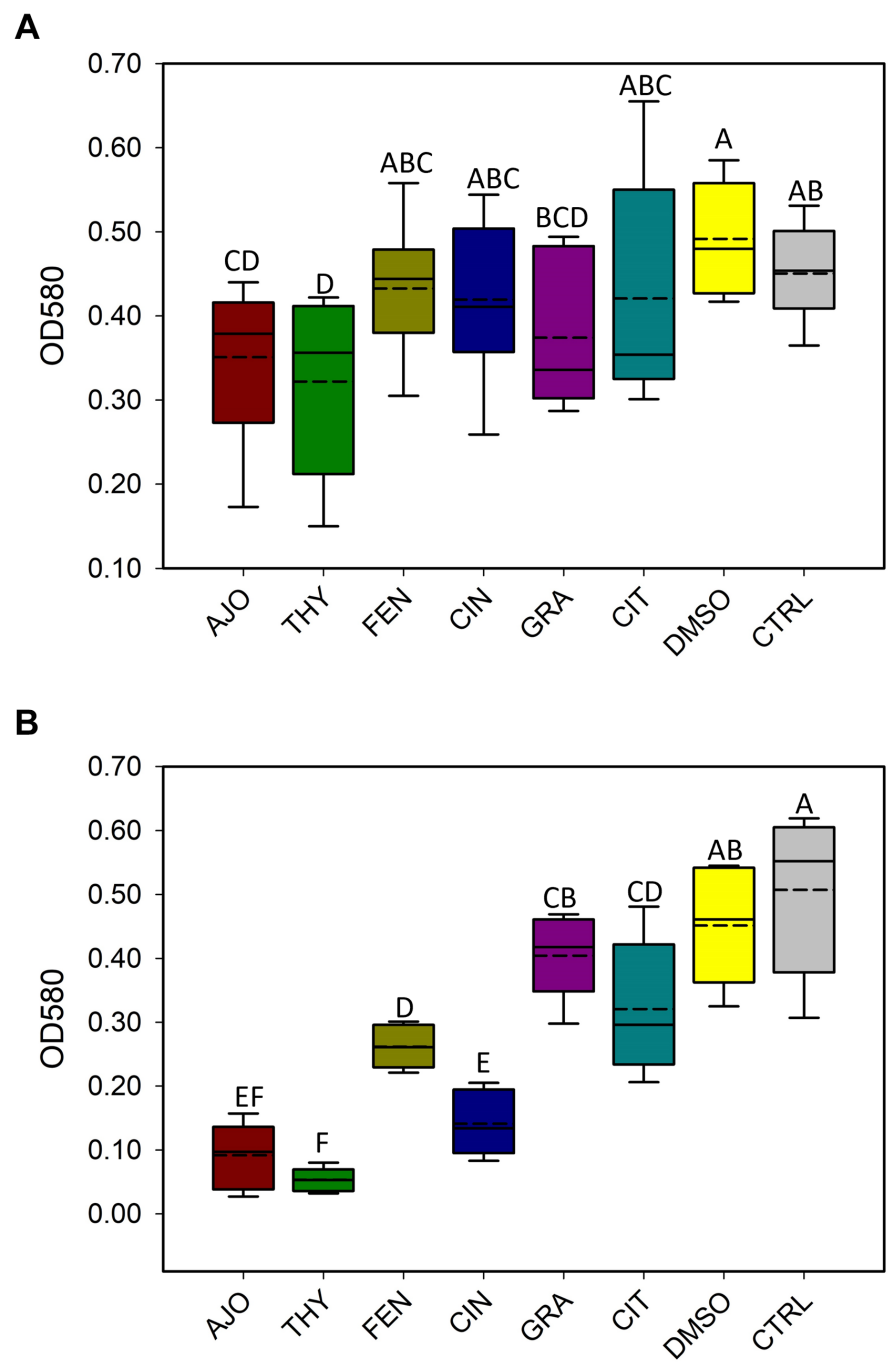
Antibiofilm activities of selected essential oils against *Escherichia coli* strain UMN026 at concentrations of **(A)** 0.025% and **(B)** 0.05% (v/v) based on optical density (OD) at 580 nm. The values are the means from four replicates. Different uppercase letters indicate significantly different means (*p* < 0.05).

With the use of CV assay, biofilms produced by *E. coli* were quantified after 48 h of incubation. Only AJO, THY, and GRA at a final concentration of 0.025% (v/v), significantly reduced (*p* < 0.05) biofilm formation by *E. coli* as compared to the DMSO and the negative control samples (Figure 9A). When the final concentration of the EOs was increased to 0.05% (v/v), all 6 EOs tested were able to significantly reduce (*p* < 0.05) the biofilm-forming capacity of *E. coli* (Figure 9). However, the most significant reduction of biofilm formation by *E. coli* was observed with AJO, THY, and CIN (Figure 9B).

### 3.5 Antiviral activity of EOs against the bovine respiratory viral pathogens

**Figure 10:**
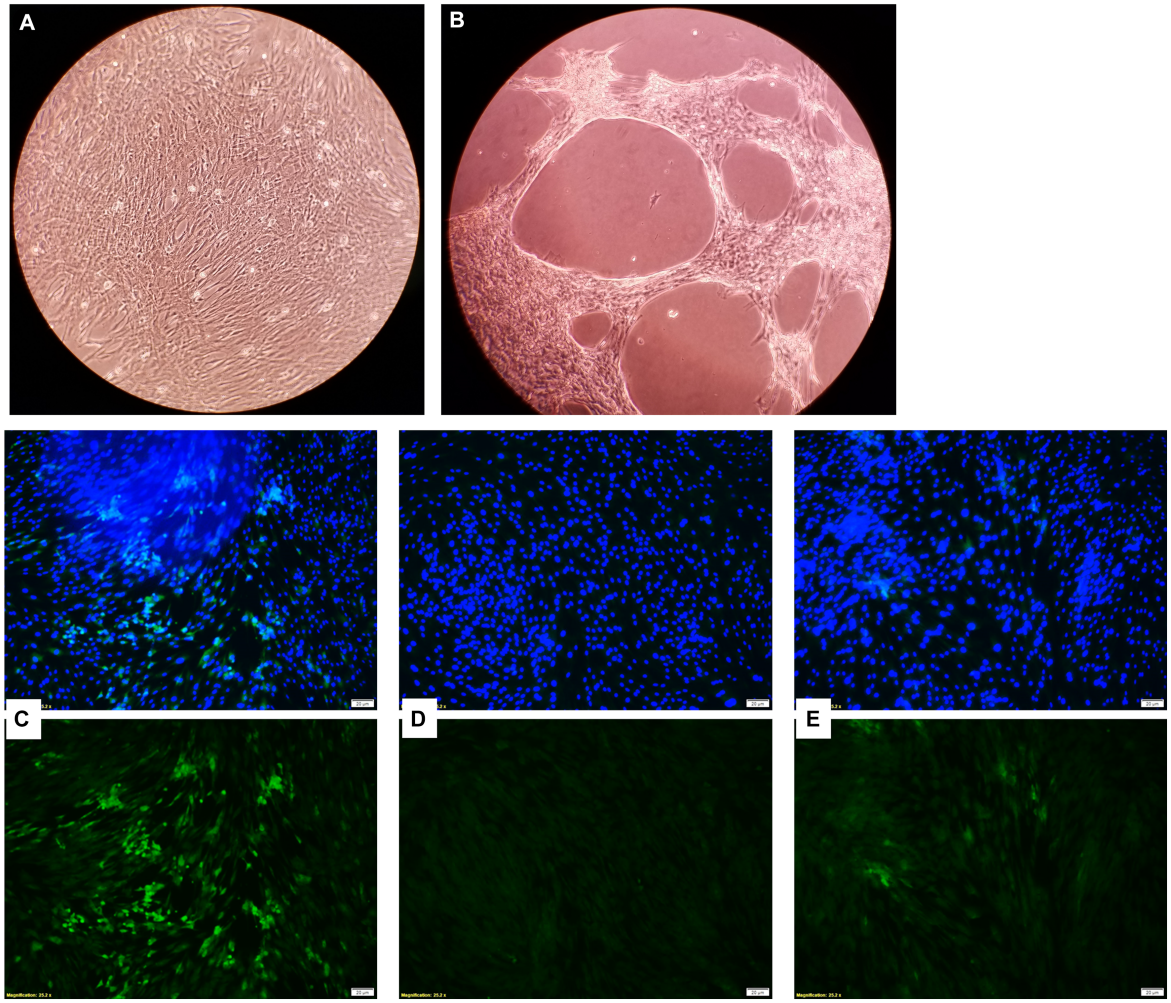
Microscopic images of bovine turbinate cells without **(A)** and with **(B)** bovine viral diarrhea virus 1 infection; Inhibition of bovine viral diarrhea virus 1 (BVDV-1) replication by essential oils. Green fluorescence is indicative of viral replication stained with FITC tagged anti-BVDV antibody and the blue fluorescence indicates the nuclei of the bovine turbinate (BT) cells stained with DAPI. **(C)** Showing the maximum viral replication on the BT cells for virus control (without EO); **(D)** Showing no viral replication on BT cells incubated DMEM control (No virus added); **(E)** Showing a minimal viral replication on BT cells incubated with THY EO (0.025%).

Bovine turbinate cells infected with BVDV-1 showed a CPE when observed under the microscope, while BT cells treated with the EOs did not and maintained a normal cytology morphology (Figures 10A,B). The EOs reduced the replication of BVDV-1 when observed through a fluorescence microscope. The fluorescein isothiocyanate (FITC) tagged anti-BVDV antibody fluorescence intensity, which represents viral replication, was the brightest in the viral positive control sample (no EOs) where the virus replicated freely in BT cells. However, the sample that received an EO in addition to the virus, had diminished fluorescence intensity, indicating a reduction in BVDV-1 replication (Figures 10C–E). Among the 15 tested EOs, the inhibitory effects on BVDV-1 replication on BT cell monolayer was observed with THY, AJO, and CIT.

## 4 Discussion

The main components of EOs can vary greatly (49) and therefore it is important to determine the exact chemical composition of individual EOs evaluated *in vitro* for reproducibility and precision. The composition of the EOs evaluated in this study was previously determined via mass spectroscopy–gas chromatography (18). Most EOs often have 1 to 3 major components (>20%) that mainly contribute to the antimicrobial activity observed with the respective EOs. As such, we selected 5 different EOs that displayed relative strong antimicrobial activities against bovine respiratory pathogens, and that they contain different main chemical components that could provide broader spectrum of antimicrobial activities against BRD pathogens when these EOs applied together. The 5 EOs tested in the present study include AJO and THY (both of which were made up of three main components such as thymol, γ-terpinene, and p-cymene), CIN (84% eugenol), CIT (38% citronellal and 23% geraniol) and FEN (78% anethole) (18). Thymol has been shown to have antimicrobial activity against many bacterial pathogens including foodborne *S. aureus* (50), *E. coli* and *Clostridium perfringens* (51), as well as clinical isolates of *Proteus mirabilis* and *Pseudomonas aeruginosa* (52), and spoilage bacteria such as *Leuconostoc citreum* (53). Along with thymol, carvacrol, trans-anethole, and 1,8 cineole EO components have been investigated for their potential use as antibacterial agents or as adjuvants for the antibiotics against *M. haemolytica* and *P. multocida* (54). While no reports on testing other pure EO components against bovine respiratory bacterial pathogens, EO components anethole (55), eugenol (56), geraniol (57), and citronellal (58) have been shown promising antibacterial activities against several human respiratory pathogens. Because of these antibacterial components, the growth of *M. haemolytica* and *P. multocida* strains were significantly inhibited by the selected AJO, CIN, FEN, and THY EOs (0.025%), as they did with different country and feedlot origin *M. haemolytica* and *P. multocida* strains tested previously (18). Overall growth of culture-enriched NS microbiota determined by OD_600_ measurement was reduced at a moderate level by AJO, THY, and FEN under both NS microbiota cultured alone or co-cultured together with *M. haemolytica*, *P. multocida* and *L. fermentum*. These results observed in culture tubes suggest that intranasal inoculation of these EOs could not only inhibit BRD bacterial pathogens but also could influence overall growth of the microbiota residing within the nasopharynx in cattle.

To further identify the impact of EOs on community structure, microbial richness, diversity, and composition of the enriched-NS microbiota, we performed 16S rRNA gene sequencing on the NS microbiota culture samples harvested at the end of 24 h incubation. Beta-diversity of the enriched-NS microbiota alone or in the presence of MPL inoculums were impacted by AJO, THY, and FEN, as shown in the distinctive clustering of these samples from DMS control, and CIN and GRA treated samples (Figure 3A). All 5 EOs (AJO, THY, FEN, CIN, and GRA) tested reduced species richness in enriched-NS microbiota, with AJO and FEN being the strongest reducers. THY was the only EO that resulted in significant alterations of community diversity, and the diversity was reduced by this EO. Overall, the addition of *M. haemolytica*, *P. multocida*, and *L. fermentum* inoculums to NS microbiota culture induced some degree of fluctuations of the EO impact on alpha diversity indices, but the significant impact of the EOs retained in NS-MPL microbiota culture. Lower microbial richness and diversity of the respiratory microbiota has been reported in cattle that developed BRD (59, 60). However, microbial richness and diversity of healthy feedlot calves were reduced in response to intranasal bacterial therapeutics comprised of bovine nasopharyngeal origin *Lactobacillus* spp. (33) as compared to the intranasal administration of saline (Control). Antibiotic tulathromycin injection, on the other hand, increased both species richness and diversity in those calves as compared to calves received intranasal bacterial therapeutics or saline (Control). The direct comparison of the impact of bacterial therapeutics and antibiotic tulathromycin suggests that reduced microbial richness and diversity of bovine upper respiratory tract may have positive association with respiratory health. As such, EO-induced microbial richness reduction in enriched-NS microbiota is most likely to be associated with positive impact on nasopharyngeal microbiota. However, this warrants further testing *in vivo*.

The cultured-enriched NS microbiota was mainly composed of *Proteobacteria*, which accounted for 99.6% of the total sequencing reads. Other phyla with lower abundance detected were *Firmicutes*, *Mycoplasmatota*, *Actinobacteria*, and *Bacteroidota*. Treatment with EOs resulted in increased abundance of *Proteobacteria* and reduced *Firmicutes* and *Bacteroidota* abundance in both enriched-NS microbiota alone or enriched-NS microbiota along with MPL. At family level, AJO, THY, and FEN EOs significantly reduced abundance of *Moraxellaceae* while promoting the growth of *Enterobacteriaceae*. An increase in *Moraxellaceae* abundance has been associated with a predisposition to BRD in cattle (57). At genus level, EO specific effect on bacterial genera was observed. Overall, the effects among the three EOs (AJO, THY, and FEN) were the same on those genera affected by EO such as *Acinetobacter* was completely suppressed, while *Escherichia* and *Klebsiella* were enriched by these three EOs. *Citrobacter* was inhibited by FEN and CIT EOs. The genera *Mannheimia, Pasteurella, Enterococcus* and *Dysgonomonas* were completely diminished by all the five EOs (AJO, THY, FEN, CIN, and GRA) tested. The variations in bacterial cellular structure and EO chemical composition could be attributed to the difference in the susceptibility of these genera to EO treatments. The effectiveness of EO on bacteria varies between Gram-positive and Gram-negative bacteria (61), and those tested EOs displayed different MICs against BRD associated pathogens and commensal bacteria such as *Lactobacillus, Bacillus*, and *Staphylococcus* spp. isolated from the nasopharynx of feedlot cattle (18). The EO specific effects on certain bacterial genera observed in this study highlights that using combination of different EOs could enhance the modulatory effects on the upper respiratory microbiota in cattle as each EO has different bacterial targets. Another important finding from these 16S rRNA gene sequencing results is that the tested EOs were able to inhibit the abundance of BRD-associated genera *Mannheimia* and *Pasteurella* in the presence of culturable nasopharyngeal microbiota. Antibiotic tolerance of a bacteria is modulated by the metabolic cross-feeding interactions between different bacteria (62). Antibiotic concentrations required to inhibit the target bacteria differ when bacteria are grown in the presence of other bacteria than in monocultures (63). Commensal bacteria could influence the virulence of opportunistic pathogens (64). Since antibiotic tolerance of a bacteria, and virulence of a pathogen are influenced by a metabolic interdependence of different bacterial species in a community, it was thus important to evaluate whether EOs can still inhibit the growth of BRD pathogens in the presence of cultivable nasopharyngeal microbiota. To the best of our knowledge, this is the first study to characterize the culturable bovine respiratory microbiota using 16S rRNA gene sequencing. The nasopharyngeal swabs were cultured in relatively less-selective media BHI, which may have limited the growth of certain bacteria that do not thrive in this media. The mean observed total ASVs in NS microbiota culture that was not treated with EO was about 30, which is less than the number of ASVs observed in nasal microbiota of newborn calves (about 150 ASVs) (65), and the ASVs (about 450 ASVs) observed from the direct sequencing of the original nasopharyngeal swabs (31) that were used in this present study.

The concentrations of most cytokines evaluated were not significantly different between EO and control groups, suggesting that EOs may have limited immune stimulatory effects on BT cell monolayers. This is an important finding as it indicates that EOs applied to upper respiratory tract would not trigger a disproportional immune response. Whether immunomodulation is beneficial depends on the intended use of the stimulant tested. As the EOs explored in this study are intended for use as an intranasal cocktail spray to mitigate BRD pathogen growth and colonization, it is preferable that they do not result in an exacerbated inflammatory response, as this can lead to tissue damage and predispose or intensify infection by opportunistic pathogens. Other EOs and their main components, such as eucalyptol (60), eugenol, and cinnamaldehyde (66), have had their immunomodulatory and anti-inflammatory properties demonstrated at lower doses. However, the applied EO dosage is critical, as higher concentrations of EOs can be cytotoxic, hepatotoxic, and/or nephrotoxic (67), highlighting the importance of demonstrating their safety *in vitro* before testing them in an *in vivo* study.

Among the 6 EOs tested, AJO, THY, and GRA reduced biofilm formation by *E. coli* strain UMN026 at 0.025% concentration, whereas CIN, CIT, FEN, and GRA displayed antibiofilm activity against *E. coli* when the concentration was 0.05%. The similar effect of AJO and THY on the biofilm formation may be expected due to their similar chemical composition, as they both have thymol, γ-terpinene, and p-cymene among their main components (18). Thus, thymol could be a potent antibiofilm compound as other studies that have used thymol alone (68) or in synergistic combination with other antimicrobial agents (69) have reported a similar effect on biofilm formation. In addition to *E. coli,* other studies have observed antibiofilm activity of EOs against *S. aureus* (70), oral cariogenic bacteria (71), and foodborne pathogens (72, 73) *in vitro*, showing the potential EOs have against persistent pathogenic biofilm formers in diverse environments. *E. coli* UMN026 was selected for this biofilm assay because bacterial BRD-associated pathogens do not normally produce biofilms; however, the NS microbiome contains biofilm-forming species, and these bacteria can influence resistance or susceptibility to the development of clinical BRD. The capacity to form biofilms by members of the respiratory tract microbiota is associated with chronic infections (74) and biofilms offer a protection barrier to these species against other bacteria occurring in the same environment. Consequently, these biofilms can also protect the host mucosa while preserving the commensal microbial diversity and persistence within the biofilms (75). There are several important human respiratory pathogens that use biofilms to persist in the respiratory tract environment and cause disease, such as *P. aeruginosa* (76). Biofilms are therefore an important bacterial defense mechanism to evade the host immune system in the respiratory tract. Neutrophils are ineffective against persistent biofilm formers (77) which also confers protection from antibiotics (78). Therefore, it was important to test the antibiofilm capacity of the selected EOs, as disrupting biofilms is an important characteristic of any substance used in the nasopharynx for BRD prevention or treatment.

Reduction of BVDV1 viral replication on BT cells was observed with THY (strong), and AJO and CIT (moderate) (0.0125%). The antiviral capacity of some EOs or EO component such as *Ocimum basilicum* and *Salvia officinalis* (79) has been demonstrated against BVDV (80, 81) and other important respiratory viral pathogens, such as influenza viruses (24, 25, 82) and respiratory syncytial virus (80) have been shown to be inhibited by EOs *in vitro*. Additionally, studies using the active compounds of EOs against clinically relevant viruses demonstrated that thymol, carvacrol, p-cymene, components of AJO and THY, and limonene, and citronellal, the major component of CIT, have antiviral activity against herpes simplex virus type 1 (83) and type 2 (84), as well as dengue virus (85) *in vitro*. The mechanism of action of EOs on viral cells is not completely elucidated but is speculated to be due to their lipophilic properties, which allow them to cross the lipid bilayer and disrupt the envelope in enveloped viruses, leading to viral inactivation. A few studies have shown antiviral activity of EOs and their constituents when tested on enteroviruses and their surrogates, although with some limitations, like the incubation temperatures (86, 87). Antiviral drugs often target viral polymerases and replication, and since EOs and their components most likely affect the envelope and capsid of viruses, this means that EOs pose minimal risk for inducing antiviral drug resistance in clinical settings.

There are a couple limitations to be acknowledged in the present study. First, using BHI to culture nasopharyngal swab associated microbiota may have limited the growth of certain bacteria that do not thrive in this media, despite BHI being non-selective medium. In addition, culturing conditions such as temperature and atmosphere, and freeze/thawing cycles could contribute to the recovery of the lower species richness in the enriched-NS microbiota. Future studies should further evaluate the impact of EOs on culturable NS microbiota *in vitro* using different culturing media, growth conditions, prolonged incubation time, and larger sample size. The second limitation is associated with antiviral assay. Due to the logistic, resources and time constraints, we were unable to proceed and enumerate the reduction of BVDV1 viral cells after treatment with the EO. Therefore, the results on antiviral activities of EOs presented in the present study were only qualitative and should be interpreted cautiously, and future quantitative assay based studies should be conducted to further confirm our results and identify the extent to which these EOs can inhibit BRD viral pathogens.

## 5 Conclusion

The EOs AJO, THY, FEN, and CIN inhibited the growth of BRD-associated pathogens *M. haemolytica* and *P. multocida* both in individual cultures and in the presence of culture-enriched NS microbiota. EOs of AJO, THY, FEN, and CIN displayed significant modulation of community structure, species richness and composition of enriched NS-microbiota. Co-culturing BT cells with AJO, THY, FEN, CIN, CIT, or GRA had minimal effect on cytokine and chemokine release from BT cells. AJO, THY, FEN, and CIN EOs demonstrated antibiofilm activity against *E. coli* UMN026. BVDV-1 viral replication in BT cell monolayer was inhibited by THY (strong), and AJO and CIT (moderate). Overall, the results of this *in vitro* study suggest that THY, AJO, CIN, CIT, FEN EOs could be used as an intranasal EO spray to modulate nasopharyngeal microbiota and mitigate BRD pathogens in feedlot cattle as an antibiotic alternative.

## Data availability statement

The raw sequencing data presented in the study can be found at the NCBI Sequence Read Archive under BioProject accession PRJNA1040875 (https://www.ncbi.nlm.nih.gov/bioproject/?term=PRJNA1040875). Other data that support the findings of this study are presented within the paper.

## Ethics statement

Ethical approval was not required for the studies on animals in accordance with the local legislation and institutional requirements because only commercially available established cell lines were used.

## Author contributions

SA: Conceptualization, Data curation, Writing – original draft, Writing – review & editing, Formal analysis, Funding acquisition, Investigation, Methodology, Project administration, Resources, Supervision, Validation, Visualization. GM: Formal analysis, Visualization, Writing – original draft, Writing – review & editing. AR: Methodology, Writing – review & editing. DH: Data curation, Formal analysis, Software, Writing – review & editing. KS: Methodology, Writing – review & editing. LK: Methodology, Writing – review & editing. SR: Methodology, Resources, Writing – review & editing.

## References

[1] Taylor JD Fulton RW Lehenbauer TW Step DL Confer AW. The epidemiology of bovine respiratory disease: What is the evidence for predisposing factors? Can Vet J. (2010) 51:1095–102. 21197200 PMC2942046

[2] Griffin D Chengappa MM Kuszak J McVey DS. Bacterial pathogens of the bovine respiratory disease complex. Vet Clin North Am Food Anim Pract. (2010) 26:381–94. doi: 10.1016/j.cvfa.2010.04.004 20619191

[3] Cusack P McMeniman N Lean I. The medicine and epidemiology of bovine respiratory disease in feedlots. Aust Vet J. (2003) 81:480–7. doi: 10.1111/j.1751-0813.2003.tb13367.x, PMID: 15086084

[4] Smith RA Step DL Woolums AR. Bovine respiratory disease: looking back and looking forward, what do we see? Vet Clin N Am Food Anim Pract. (2020) 36:239–51. doi: 10.1016/j.cvfa.2020.03.009, PMID: 32451026

[5] Zeineldin M Lowe J Aldridge B. Contribution of the mucosal microbiota to bovine respiratory health. Trends Microbiol. (2019) 27:753–70. doi: 10.1016/j.tim.2019.04.005, PMID: 31104970

[6] Hilton WM. BRD in 2014: where have we been, where are we now, and where do we want to go? Anim Health Res Rev. (2014) 15:120–2. doi: 10.1017/S1466252314000115, PMID: 25358813

[7] Edwards TA. Control methods for bovine respiratory disease for feedlot cattle. Vet Clin N Am Food Anim Pract. (2010) 26:273–84. doi: 10.1016/j.cvfa.2010.03.005 20619184

[8] Vogel GJ Bokenkroger CD Rutten-Ramos SC Bargen JL. A Retrospective evaluation of animal mortality in US feedlots: Rate, timing, and cause of death. Bovine Practitioner. (2015) 49:113–23. doi: 10.21423/bovine-vol49no2p113-123

[9] Klima CL Zaheer R Cook SR Booker CW Hendrick S Alexander TW. Pathogens of bovine respiratory disease in North American feedlots conferring multidrug resistance via integrative conjugative elements. J Clin Microbiol. (2014) 52:438–48. doi: 10.1128/JCM.02485-13, PMID: 24478472 PMC3911356

[10] Anholt RM Klima C Allan N Matheson-Bird H Schatz C Ajitkumar P. Antimicrobial susceptibility of bacteria that cause bovine respiratory disease complex in Alberta, Canada. Front Vet Sci. (2017) 4:207. doi: 10.3389/fvets.2017.00207, PMID: 29255716 PMC5723070

[11] Snyder E Credille B Berghaus R Giguère S. Prevalence of multi drug antimicrobial resistance in isolated from high-risk stocker cattle at arrival and two weeks after processing. J Anim Sci. (2017) 95:1124–31. doi: 10.2527/JAS.2016.1110, PMID: 28380515

[12] Timsit E Hallewell J Booker C Tison N Amat S Alexander TW. Prevalence and antimicrobial susceptibility of *Mannheimia haemolytica*, Pasteurella multocida, and *Histophilus somni* isolated from the lower respiratory tract of healthy feedlot cattle and those diagnosed with bovine respiratory disease. Vet Microbiol. (2017) 208:118–25. doi: 10.1016/j.vetmic.2017.07.013 28888626

[13] Amat S Alexander TW Holman DB Schwinghamer T Timsit E. intranasal bacterial therapeutics reduce colonization by the respiratory pathogen *Mannheimia haemolytica* in dairy calves. mSystems. (2020) 5:e00629-19. doi: 10.1128/mSystems.00629-19, PMID: 32127421 PMC7055656

[14] Burt S. Essential oils: their antibacterial properties and potential applications in foods—a review. Int J Food Microbiol. (2004) 94:223–53. doi: 10.1016/j.ijfoodmicro.2004.03.022, PMID: 15246235

[15] Hammer KA Carson CF Riley TV. Antimicrobial activity of essential oils and other plant extracts. J Appl Microbiol. (1999) 86:985–90. doi: 10.1046/j.1365-2672.1999.00780.x 10438227

[16] Yap PSX Yiap BC Ping HC Lim SHE. Essential oils, a new horizon in combating bacterial antibiotic resistance. Open Microbiol J. (2014) 8:6–14. doi: 10.2174/1874285801408010006, PMID: 24627729 PMC3950955

[17] Amat S Subramanian S Timsit E Alexander TW. Probiotic bacteria inhibit the bovine respiratory pathogen *Mannheimia haemolytica* serotype 1 *in vitro*. Lett Appl Microbiol. (2017) 64:343–9. doi: 10.1111/LAM.12723, PMID: 28178767

[18] Amat S Baines D Timsit E Hallewell J Alexander TW. Essential oils inhibit the bovine respiratory pathogens *Mannheimia haemolytica*, Pasteurella multocida and Histophilus somni and have limited effects on commensal bacteria and turbinate cells *in vitro*. J Appl Microbiol. (2019) 126:1668–82. doi: 10.1111/JAM.14238, PMID: 30817050

[19] Bismarck D Becker J Müller E Becher V Nau L Mayer P. Screening of antimicrobial activity of essential oils against bovine respiratory pathogens – focusing on *Pasteurella multocida*. Planta Med. (2022) 88:274–81. doi: 10.1055/a-1726-9291, PMID: 35180782 PMC8967432

[20] Yap PSX Lim SHE Hu CP Yiap BC. Combination of essential oils and antibiotics reduce antibiotic resistance in plasmid-conferred multidrug resistant bacteria. Phytomedicine. (2013) 20:710–3. doi: 10.1016/J.PHYMED.2013.02.013, PMID: 23537749

[21] Knezevic P Aleksic V Simin N Svircev E Petrovic A Mimica-Dukic N. Antimicrobial activity of *Eucalyptus camaldulensis* essential oils and their interactions with conventional antimicrobial agents against multi-drug resistant *Acinetobacter baumannii*. J Ethnopharmacol. (2016) 178:125–36. doi: 10.1016/j.jep.2015.12.008, PMID: 26671210

[22] Basavegowda N Patra JK Baek K-H. Essential oils and Mono/bi/tri-metallic nanocomposites as alternative sources of antimicrobial agents to combat multidrug-resistant pathogenic microorganisms: an overview. Molecules. (2020) 25:1058. doi: 10.3390/molecules25051058, PMID: 32120930 PMC7179174

[23] Ragno R Papa R Patsilinakos A Vrenna G Garzoli S Tuccio V. Essential oils against bacterial isolates from cystic fibrosis patients by means of antimicrobial and unsupervised machine learning approaches. Sci Rep. (2020) 10:2653. doi: 10.1038/s41598-020-59553-8, PMID: 32060344 PMC7021809

[24] Wu S Patel KB Booth LJ Metcalf JP Lin H-K Wu W. Protective essential oil attenuates influenza virus infection: An *in vitro* study in MDCK cells. BMC Complement Altern Med. (2010) 10:69. doi: 10.1186/1472-6882-10-69, PMID: 21078173 PMC2994788

[25] Choi HJ. Chemical constituents of essential oils possessing anti-influenza A/WS/33 virus activity. Osong Public Health Res Perspect. (2018) 9:348–53. doi: 10.24171/J.PHRP.2018.9.6.09, PMID: 30584499 PMC6296812

[26] Asif M Saleem M Saadullah M Yaseen HS al Zarzour R. COVID-19 and therapy with essential oils having antiviral, anti-inflammatory, and immunomodulatory properties. Inflammopharmacol. (2020) 28:1153–61. doi: 10.1007/s10787-020-00744-0, PMID: 32803479 PMC7427755

[27] Brochot A Guilbot A Haddioui L Roques C. Antibacterial, antifungal, and antiviral effects of three essential oil blends. MicrobiologyOpen. (2017) 6:e00459. doi: 10.1002/mbo3.459, PMID: 28296357 PMC5552930

[28] Gómez-Sequeda N Cáceres M Stashenko EE Hidalgo W Ortiz C. Antimicrobial and antibiofilm activities of essential oils against *Escherichia coli* O157:H7 and methicillin-resistant *Staphylococcus aureus* (MRSA). Antibiotics. (2020) 9:730. doi: 10.3390/antibiotics9110730, PMID: 33114324 PMC7690905

[29] Liu T Wang J Gong X Wu X Liu L Chi F. Rosemary and tea tree essential oils exert antibiofilm activities *in vitro* against staphylococcus aureus and *Escherichia coli*. J Food Prot. (2020) 83:1261–7. doi: 10.4315/0362-028X.JFP-19-337, PMID: 32577759

[30] Sandner G Heckmann M Weghuber J. Immunomodulatory activities of selected essential oils. Biomol Ther. (2020) 10:1139. doi: 10.3390/BIOM10081139, PMID: 32756359 PMC7464830

[31] Winders TM Holman DB Schmidt KN Luecke SM Smith DJ Neville BW. Feeding hempseed cake alters the bovine gut, respiratory and reproductive microbiota. Sci Rep. (2023) 13:8121. doi: 10.1038/s41598-023-35241-1, PMID: 37208436 PMC10199011

[32] Amat S Timsit E Baines D Yanke J Alexander TW. Development of bacterial therapeutics against the bovine respiratory pathogen *Mannheimia haemolytica*. Appl Environ Microbiol. (2019) 85:e01359-19. doi: 10.1128/AEM.01359-19, PMID: 31444198 PMC6803296

[33] Amat S Timsit E Workentine M Schwinghamer T Van Der Meer F Guo Y. A single intranasal dose of bacterial therapeutics to calves confers longitudinal modulation of the nasopharyngeal microbiota: a pilot study. Microbial systems. (2023) 8:e01016–22. doi: 10.1128/msystems.01016-22, PMID: 36971568 PMC10134831

[34] Amat S Holman DB Schmidt K Menezes ACB Baumgaertner F Winders T. The nasopharyngeal, ruminal, and vaginal microbiota and the core taxa shared across these microbiomes in virgin yearling heifers exposed to divergent in utero nutrition during their first trimester of gestation and in pregnant beef heifers in response to mineral supplementation. Microorganisms. (2021) 9:2011. doi: 10.3390/microorganisms9102011, PMID: 34683332 PMC8537542

[35] Santiago-Rodriguez TM Fornaciari G Luciani S Dowd SE Toranzos GA Marota I. Taxonomic and predicted metabolic profiles of the human gut microbiome in pre-Columbian mummies. FEMS Microbiol Ecol. (2016) 92:fiw182. doi: 10.1093/femsec/fiw182, PMID: 27559027

[36] Callahan BJ McMurdie PJ Rosen MJ Han AW Johnson AJA Holmes SP. (2016). DADA2: High-resolution sample inference from Illumina amplicon data. Nature Methods 13: 13:581–583. doi: 10.1038/nmeth.3869 27214047 PMC4927377

[37] Quast C Pruesse E Yilmaz P Gerken J Schweer T Yarza P. The SILVA ribosomal RNA gene database project: improved data processing and web-based tools. Nucleic Acids Res. (2013) 41:D590–6. doi: 10.1093/nar/gks1219, PMID: 23193283 PMC3531112

[38] Wang Q Garrity GM Tiedje JM Cole JR. Naïve Bayesian classifier for rapid assignment of rRNA sequences into the new bacterial taxonomy. Appl Environ Microbiol. (2007) 73:5261–7. doi: 10.1128/AEM.00062-07, PMID: 17586664 PMC1950982

[39] McMurdie PJ Holmes S. phyloseq: An R package for reproducible interactive analysis and graphics of microbiome census data. PLoS One. (2013) 8:e61217. doi: 10.1371/journal.pone.0061217, PMID: 23630581 PMC3632530

[40] Oskanen J Kindt R O’Hara R. vegan: Community Ecology Package. (2012).Available at: https://cir.nii.ac.jp/crid/1571980076147214336 (Accessed May 27, 2023).

[41] O’Toole GA. Microtiter dish biofilm formation assay. J Vis Exp. (2011) 47:2437. doi: 10.3791/2437, PMID: 21307833 PMC3182663

[42] Neidhardt FC Bloch PL Smith DF. Culture medium for enterobacteria. J Bacteriol. (1974) 119:736–47. doi: 10.1128/jb.119.3.736-747.1974, PMID: 4604283 PMC245675

[43] Shahriar FM Clark EG Janzen E West K Wobeser G. Coinfection with bovine viral diarrhea virus and *Mycoplasma bovis* in feedlot cattle with chronic pneumonia. Can Vet J. (2002) 43:863–8. PMID: 12497963 PMC339759

[44] Bürgi N Josi C Bürki S Schweizer M Pilo P. *Mycoplasma bovis* co-infection with bovine viral diarrhea virus in bovine macrophages. Vet Res. (2018) 49:2. doi: 10.1186/s13567-017-0499-1, PMID: 29316971 PMC5761114

[45] Reed LJ Muench H. A simple method of estimating fifty per cent endpoints12. Am J Epidemiol. (1938) 27:493–7. doi: 10.1093/oxfordjournals.aje.a118408

[46] Bonifaz LC Arzate S Moreno J. Endogenous and exogenous forms of the same antigen are processed from different pools to bind MHC class II molecules in endocytic compartments. Eur J Immunol. (1999) 29:119–31. doi: 10.1002/(SICI)1521-4141(199901)29:01<119::AID-IMMU119>3.0.CO;2-O, PMID: 9933093

[47] Rakibuzzaman AGM Kolyvushko O Singh G Nara P Piñeyro P Leclerc E. Targeted alteration of antibody-based immunodominance enhances the heterosubtypic immunity of an experimental PCV2 vaccine. Vaccine. (2020) 8:506. doi: 10.3390/vaccines8030506, PMID: 32899842 PMC7563983

[48] Rakibuzzaman A Piñeyro P Pillatzki A Ramamoorthy S. Harnessing the Genetic plasticity of porcine circovirus type 2 to target suicidal replication. Viruses. (2021) 13:1676. doi: 10.3390/v13091676, PMID: 34578257 PMC8473201

[49] Teixeira B Marques A Ramos C Serrano C Matos O Neng NR. Chemical composition and bioactivity of different oregano (*Origanum vulgare*) extracts and essential oil. J Sci Food Agric. (2013) 93:2707–14. doi: 10.1002/jsfa.6089, PMID: 23553824

[50] Rúa J del Valle P de Arriaga D Fernández-Álvarez L García-Armesto MR. Combination of carvacrol and thymol: antimicrobial activity against staphylococcus aureus and antioxidant activity. Foodborne Pathog Dis. (2019) 16:622–9. doi: 10.1089/fpd.2018.2594, PMID: 31009261

[51] Sepahvand S Amiri S Radi M Akhavan H-R. Antimicrobial activity of thymol and thymol-nanoemulsion against three food-borne pathogens inoculated in a sausage model. Food Bioprocess Technol. (2021) 14:1936–45. doi: 10.1007/s11947-021-02689-w

[52] Sim JXF Khazandi M Chan WY Trott DJ Deo P. Antimicrobial activity of thyme oil, oregano oil, thymol and carvacrol against sensitive and resistant microbial isolates from dogs with otitis externa. Vet Dermatol. (2019) 30:524–e159. doi: 10.1111/vde.12794, PMID: 31566822

[53] Lee S Kim H Beuchat LR Kim Y Ryu J-H. Synergistic antimicrobial activity of oregano and thyme thymol essential oils against *Leuconostoc citreum* in a laboratory medium and tomato juice. Food Microbiol. (2020) 90:103489:103489. doi: 10.1016/j.fm.2020.103489, PMID: 32336377

[54] Kissels W Wu X Santos RR. Short communication: Interaction of the isomers carvacrol and thymol with the antibiotics doxycycline and tilmicosin: *In vitro* effects against pathogenic bacteria commonly found in the respiratory tract of calves. J Dairy Sci. (2017) 100:970–4. doi: 10.3168/JDS.2016-11536, PMID: 28012625

[55] Kwiatkowski P Pruss A Masiuk H Mnichowska-Polanowska M Kaczmarek M Giedrys-Kalemba S. The effect of fennel essential oil and trans-anethole on antibacterial activity of mupirocin against *Staphylococcus aureus* isolated from asymptomatic carriers. Postepy Dermatol Alergol. (2019) 36:308–14. doi: 10.5114/ada.2018.76425, PMID: 31333348 PMC6640024

[56] Yadav MK Chae S-W Im GJ Chung J-W Song J-J. Eugenol: a phyto-compound effective against methicillin-resistant and methicillin-sensitive *Staphylococcus aureus* clinical strain biofilms. PLoS One. (2015) 10:e0119564. doi: 10.1371/journal.pone.0119564, PMID: 25781975 PMC4364371

[57] Mączka W Wińska K Grabarczyk M. One hundred faces of geraniol. Mol Ther. (2020) 25:3303. doi: 10.3390/molecules25143303, PMID: 32708169 PMC7397177

[58] Ács K Balázs VL Kocsis B Bencsik T Böszörményi A Horváth G. Antibacterial activity evaluation of selected essential oils in liquid and vapor phase on respiratory tract pathogens. BMC Complement Altern Med. (2018) 18:227. doi: 10.1186/s12906-018-2291-9, PMID: 30053847 PMC6064118

[59] Holman DB McAllister TA Topp E Wright ADG Alexander TW. The nasopharyngeal microbiota of feedlot cattle that develop bovine respiratory disease. Vet Microbiol. (2015) 180:90–5. doi: 10.1016/J.VETMIC.2015.07.031, PMID: 26249828

[60] Yin C Liu B Wang P Li X Li Y Zheng X. Eucalyptol alleviates inflammation and pain responses in a mouse model of gout arthritis. Br J Pharmacol. (2020) 177:2042–57. doi: 10.1111/bph.14967, PMID: 31883118 PMC7161556

[61] Prabuseenivasan S Jayakumar M Ignacimuthu S. *In vitro* antibacterial activity of some plant essential oils. BMC Complement Altern Med. (2006) 6:39. doi: 10.1186/1472-6882-6-39, PMID: 17134518 PMC1693916

[62] Adamowicz EM Flynn J Hunter RC Harcombe WR. Cross-feeding modulates antibiotic tolerance in bacterial communities. ISME J. (2018) 12:2723–35. doi: 10.1038/s41396-018-0212-z, PMID: 29991761 PMC6194032

[63] Adamowicz EM Muza M Chacón JM Harcombe WR. Cross-feeding modulates the rate and mechanism of antibiotic resistance evolution in a model microbial community of Escherichia coli and *Salmonella enterica*. PLoS Pathog. (2020) 16:e1008700. doi: 10.1371/journal.ppat.1008700, PMID: 32687537 PMC7392344

[64] Stacy A Fleming D Lamont RJ Rumbaugh KP Whiteley M. A commensal bacterium promotes virulence of an opportunistic pathogen via cross-respiration. M bio. (2016) 7:e00782–16. doi: 10.1128/mBio.00782-16, PMID: 27353758 PMC4916382

[65] Luecke SM Holman DB Schmidt KN Gzyl KE Hurlbert JL Menezes ACB. Whole-body microbiota of newborn calves and their response to prenatal vitamin and mineral supplementation. Front Microbiol. (2023) 14:1207601. doi: 10.3389/fmicb.2023.1207601, PMID: 37434710 PMC10331429

[66] Mateen S Rehman MT Shahzad S Naeem SS Faizy AF Khan AQ. Anti-oxidant and anti-inflammatory effects of cinnamaldehyde and eugenol on mononuclear cells of rheumatoid arthritis patients. Eur J Pharmacol. (2019) 852:14–24. doi: 10.1016/j.ejphar.2019.02.031, PMID: 30796902

[67] Janes SEJ Price CSG Thomas D. Essential oil poisoning: N-acetylcysteine for eugenol-induced hepatic failure and analysis of a national database. Eur J Pediatr. (2005) 164:520–2. doi: 10.1007/s00431-005-1692-1, PMID: 15895251

[68] Čabarkapa I Čolović R Đuragić O Popović S Kokić B Milanov D. Anti-biofilm activities of essential oils rich in carvacrol and thymol against *Salmonella Enteritidis*. Biofouling. (2019) 35:361–75. doi: 10.1080/08927014.2019.1610169, PMID: 31088182

[69] Hossain MI Rahaman Mizan MF Toushik SH Roy PK Jahid IK Park SH. Antibiofilm effect of nisin alone and combined with food-grade oil components (thymol and eugenol) against *Listeria monocytogenes* cocktail culture on food and food-contact surfaces. Food Control. (2022) 135:108796. doi: 10.1016/j.foodcont.2021.108796

[70] Budzyńska A Wieckowska-Szakiel M Sadowska B Kalemba D Rózalska B. Antibiofilm activity of selected plant essential oils and their major components. Pol J Microbiol. (2011) 60:35–41. doi: 10.33073/pjm-2011-005, PMID: 21630572

[71] de Oliveira Carvalho I Purgato GA Píccolo MS Pizziolo VR Coelho RR Diaz-Muñoz G. *In vitro* anticariogenic and antibiofilm activities of toothpastes formulated with essential oils. Arch Oral Biol. (2020) 117:104834. doi: 10.1016/j.archoralbio.2020.104834, PMID: 32663696

[72] Snoussi M Dehmani A Noumi E Flamini G Papetti A. Chemical composition and antibiofilm activity of *Petroselinum crispum* and *Ocimum basilicum* essential oils against *Vibrio* spp. strains. Microb Pathog. (2016) 90:13–21. doi: 10.1016/j.micpath.2015.11.004 26596707

[73] Mouhoub A Guendouz A Belkamel A el Alaoui Talibi Z Ibnsouda Koraichi S el Modafar C. Assessment of the antioxidant, antimicrobial and antibiofilm activities of essential oils for potential application of active chitosan films in food preservation. World J Microbiol Biotechnol. (2022) 38:179. doi: 10.1007/s11274-022-03363-9, PMID: 35941332

[74] Bjarnsholt T. The role of bacterial biofilms in chronic infections. APMIS. (2013) 121:1–58. doi: 10.1111/apm.12099 23635385

[75] Burmølle M Thomsen TR Fazli M Dige I Christensen L Homøe P. Biofilms in chronic infections - a matter of opportunity - monospecies biofilms in multispecies infections. FEMS Immunol Med Microbiol. (2010) 59:324–36. doi: 10.1111/j.1574-695X.2010.00714.x, PMID: 20602635

[76] Parsek MR Tolker-Nielsen T. Pattern formation in *Pseudomonas aeruginosa* biofilms. Curr Opin Microbiol. (2008) 11:560–6. doi: 10.1016/j.mib.2008.09.015 18935979

[77] Yonker LM Cigana C Hurley BP Bragonzi A. Host-pathogen interplay in the respiratory environment of cystic fibrosis. J Cyst Fibros. (2015) 14:431–9. doi: 10.1016/j.jcf.2015.02.008, PMID: 25800687 PMC4485938

[78] Drenkard E. Antimicrobial resistance of *Pseudomonas aeruginosa* biofilms. Microbes Infect. (2003) 5:1213–9. doi: 10.1016/j.micinf.2003.08.009 14623017

[79] Madeddu S Marongiu A Sanna G Zannella C Falconieri D Porcedda S. Bovine viral diarrhea virus (BVDV): a preliminary study on antiviral properties of some aromatic and medicinal plants. Pathogens. (2021) 10:403. doi: 10.3390/pathogens10040403 33805453 PMC8066157

[80] Pilau MR Alves SH Weiblen R Arenhart S Cueto AP Lovato LT. Antiviral activity of the *Lippia graveolens* (Mexican oregano) essential oil and its main compound carvacrol against human and animal viruses. Braz J Microbiol. (2011) 42:1616–24. doi: 10.1590/S1517-83822011000400049, PMID: 24031796 PMC3768712

[81] Kubiça TF Alves SH Weiblen R Lovato LT. *In vitro* inhibition of the bovine viral diarrhoea virus by the essential oil of *Ocimum basilicum* (basil) and monoterpenes. Braz J Microbiol. (2014) 45:209–14. doi: 10.1590/S1517-83822014005000030, PMID: 24948933 PMC4059298

[82] Vimalanathan S Hudson J. Anti-influenza virus activity of essential oils and vapors. Am J Essent Oils Nat Products. (2014) 2:47–53.

[83] Sharifi-Rad J Salehi B Schnitzler P Ayatollahi SA Kobarfard F Fathi M. Susceptibility of herpes simplex virus type 1 to monoterpenes thymol, carvacrol, p-cymene and essential oils of *Sinapis arvensis* L., Lallemantia royleana Benth. and Pulicaria vulgaris Gaertn. Cell Mol Biol (Noisy-le-Grand). (2017) 63:42–7. doi: 10.14715/cmb/2017.63.8.10, PMID: 28886313

[84] Allahverdiyev A Duran N Ozguven M Koltas S. Antiviral activity of the volatile oils of *Melissa officinalis* L. against Herpes simplex virus type-2. Phytomedicine. (2004) 11:657–61. doi: 10.1016/j.phymed.2003.07.014 15636181

[85] Nagy MM Al-Mahdy DA Abd El Aziz OM Kandil AM Tantawy MA El Alfy TSM. Chemical composition and antiviral activity of essential oils from *Citrus reshni* hort. ex Tanaka (*Cleopatra mandarin*) cultivated in Egypt. J Essent Oil Bearing Plants. (2018) 21:264–72. doi: 10.1080/0972060X.2018.1436986

[86] Gilling D Kitajima M Torrey JR Bright KR. Antiviral efficacy and mechanisms of action of oregano essential oil and its primary component carvacrol against murine norovirus. J Appl Microbiol. (2014) 116:1149–63. doi: 10.1111/jam.12453, PMID: 24779581

[87] Tait S Salvati AL Desideri N Fiore L. Antiviral activity of substituted homoisoflavonoids on enteroviruses. Antivir Res. (2006) 72:252–5. doi: 10.1016/j.antiviral.2006.07.003, PMID: 16934879

